# Fluorine-containing FDA-approved small-molecule drugs in 2025: significance, synthetic insights, and therapeutic applications

**DOI:** 10.1039/d6ra01711h

**Published:** 2026-03-13

**Authors:** Shweta Mishra, Chetna Jadala, Savio Cardoza, Gal Reddy Potuganti, Ganga Reddy Velma

**Affiliations:** a Dr. D.Y. Patil Institute of Pharmaceutical Sciences and Research Sant Tukaram Nagar, Pimpri Pune 411018 India; b DPGU-School of Pharmacy and Research Sant Tukaram Nagar, Pimpri Pune 411018 India; c Indiana University School of Medicine, Indiana University (IU) Indianapolis IN 46202 USA; d Department of Pharmacology and Toxicology, College of Pharmacy, University of Arizona Tucson AZ 85721 USA vgreddy@arizona.edu velmagangareddy47@gmail.com; e Department of Chemistry and Biochemistry, The University of Oklahoma Norman Oklahoma 73019 USA

## Abstract

Fluorine has been widely incorporated into small-molecule drugs as an effective strategy to modulate metabolic stability, lipophilicity, target interactions, and overall pharmacokinetic profile. As a result, fluorine-containing compounds remain well represented among newly approved therapeutics across a broad spectrum of disease areas; notably, in 2025, 14 of the 29 small-molecule drugs approved by the U.S. Food and Drug Administration (FDA) contained at least one fluorine atom, underscoring the continued relevance of fluorine in contemporary drug design. This annual review highlights the role of fluorine in modern medicinal chemistry by summarizing FDA-approved fluorine-containing small-molecule drugs in 2025. The review examines their therapeutic indications, molecular targets, and structural features, and outlines representative synthetic strategies used in their preparation. Overall, this review provides an updated perspective on the continued importance of fluorine chemistry in modern drug discovery and development.

## Abstract 2

Fluorine has been widely incorporated into small-molecule drugs as an effective strategy to modulate metabolic stability, lipophilicity, target interactions, and overall pharmacokinetic profile.

## Graphical abstract

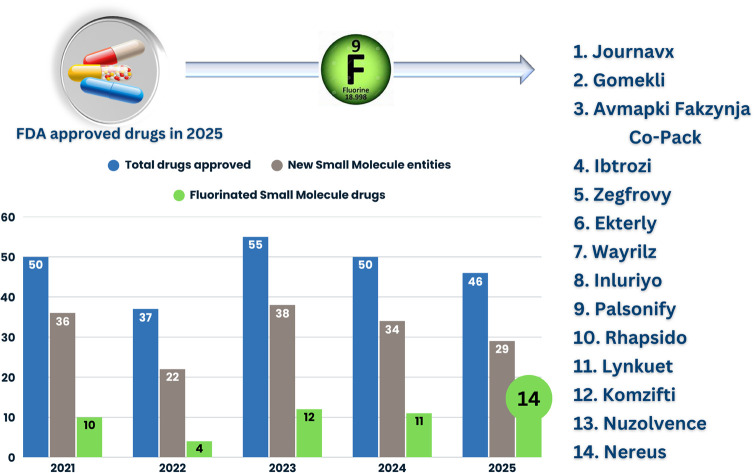

## 1 Introduction

Fluorine plays a central role in modern medicinal chemistry due to its distinctive physicochemical properties and its ability to fine-tune drug behavior at the molecular level.^1^ Incorporation of fluorine into small-molecule therapeutics is widely used to improve potency, metabolic stability, and pharmacokinetic performance without imposing substantial steric demand.^2^ The strong carbon–fluorine bond limits metabolic oxidation, often leading to enhanced *in vivo* stability and reduced formation of undesired metabolites.^3–5^ At the same time, fluorine's high electronegativity influences electronic distribution, lipophilicity, and ionization equilibria, thereby modulating membrane permeability and target binding.^1^ Owing to these features, fluorine is frequently employed as a bioisostere for hydrogen, alkyl, or heteroatom-containing functionalities, enabling precise control of molecular interactions.^5,6^ Beyond its impact on drug design, fluorine also holds unique value in biomedical imaging, with ^19^F NMR and ^18^F-based positron emission tomography (PET) serving as important tools for diagnostics and translational research.^7^ Current estimates suggest that roughly 15–20% of marketed small-molecule drugs incorporate at least one fluorine atom, reflecting the routine application of this approach in modern drug discovery.^8,9^

**Fig. 1:**
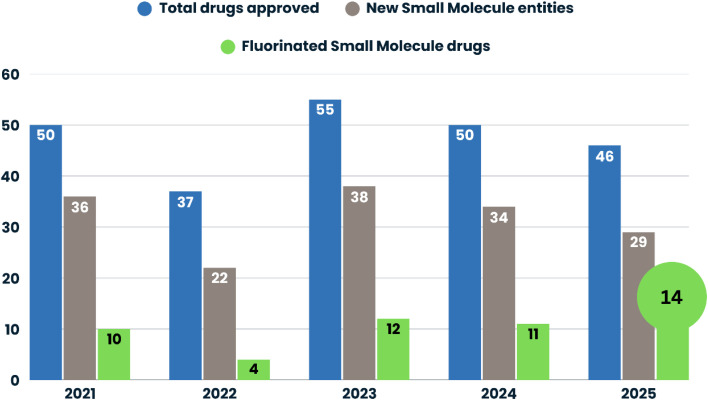
Trends in FDA drug approvals and fluorine-containing small molecule drugs from 2021 to 2025.

Fluorinated small molecules account for a substantial proportion of recent U.S. FDA approvals.^10,11^ In 2025, 14 of the 29 newly approved small-molecule entities (approximately 48%) contained at least one fluorine atom, representing the highest proportion observed over the 2021–2025 period (Fig. 1). While the total number of approvals fluctuated during these years, the relative contribution of fluorinated agents has remained consistently significant, with a marked resurgence following the dip in 2022. This sustained prevalence highlights not only the routine incorporation of fluorine in modern drug design but also its strategic deployment across diverse therapeutic classes in 2025, including kinase inhibitors, receptor and enzyme modulators, ion channel blockers, and anti-infective agents. The data further suggest that fluorination remains a key tool for optimizing pharmacokinetic performance, metabolic stability, and target selectivity, reinforcing its central role in contemporary medicinal chemistry and translational drug development.^7^

**Fig. 2:**
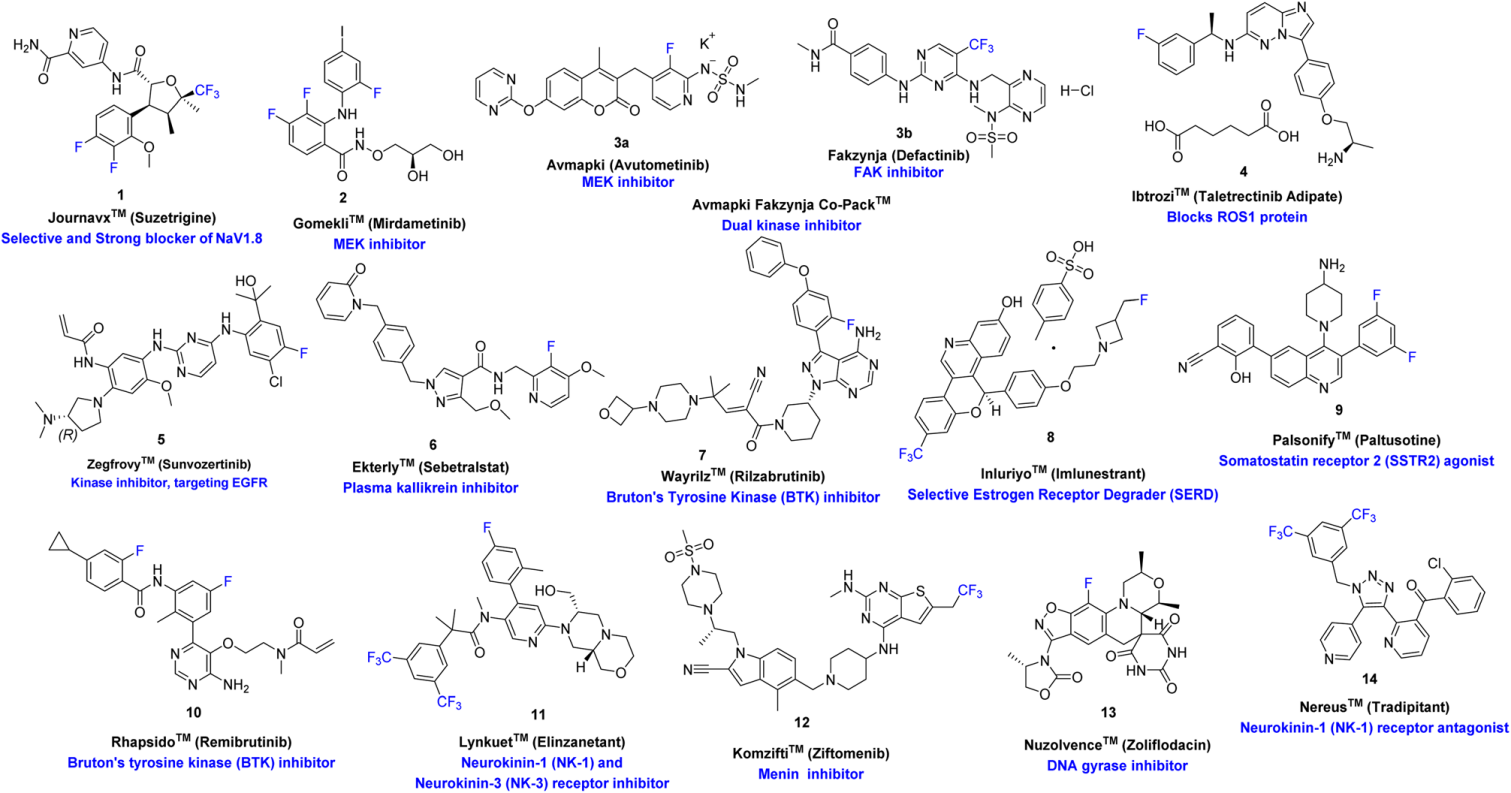
Fluorine-containing drugs approved by the U.S FDA in 2025.

The fluorine-containing small-molecule drugs approved by the U.S. FDA in 2025 span a broad range of therapeutic targets and disease indications (Fig. 2).^12^ Among these are kinase-targeted therapies such as Gomekli™ (2, Mirdametinib), a MEK inhibitor for oncological indications; Avmapki™ (3a, Avutometinib), also a MEK inhibitor approved in combination with Fakzynja™ (3b, Defactinib), a focal adhesion kinase (FAK) inhibitor, as the Avmapki Fakzynja Co-Pack™, providing a dual-kinase inhibition strategy; Ibtrozi™ (4, taletrectinib adipate), a ROS1 inhibitor for advanced malignancies; Zegfrovy™ (5, sunvozertinib), an EGFR inhibitor; and the Bruton's tyrosine kinase (BTK) inhibitors Wayrilz™ (7, rilzabrutinib) and Rhapsido™ (10, remibrutinib). Beyond kinase inhibition, other mechanistic classes are represented, including Journavx™ (1, suzetrigine), a selective blocker of the voltage-gated sodium channel NaV1.8 for pain management; Ekterly™ (6, sebetralstat), a plasma kallikrein inhibitor; Inluriyo™ (8, imlunestrant), a selective estrogen receptor degrader (SERD); and Palsonify™ (9, paltusotine), a somatostatin receptor-2 (SSTR2) agonist. Neurokinin-targeted therapies include Lynkuet™ (11, elinzanetant), a dual NK-1/NK-3 receptor antagonist, and Nereus™ (14, tradipitant), a selective NK-1 receptor antagonist. Additionally, Komzifti™ (12, ziftomenib) was approved as a menin inhibitor for hematological malignancies, while Nuzolvence™ (13, zoliflodacin) represents a first-in-class DNA gyrase inhibitor for the treatment of bacterial infections. Across these agents, fluorine substitution, ranging from monofluoro substituents to trifluoromethyl motifs, contributes to enhanced target affinity, metabolic resilience, and overall pharmacological performance across diverse therapeutic contexts.^3–5^

Over successive decades, approvals of fluorinated drugs have increased steadily, and multiple reviews have documented progress in this area.^10,11,13,14^ The recent annual two reviews summarized fluorine-containing FDA-approved small-molecule drugs in 2024,^7^ highlighting the continued prominence of fluorination strategies in approved therapeutics. Building on these year-specific analyses, the present review focuses exclusively on FDA approvals in 2025, a cohort characterized by distinct therapeutic distributions, structural motifs, and molecular targets. Table 1 provides a structured overview of the 2025 approvals, including active pharmaceutical ingredients, therapeutic indications, recommended dosages, and approval dates, thereby establishing the regulatory and clinical context for the subsequent comparative synthetic and medicinal chemistry discussion. Rather than serving solely as an annual catalog, this manuscript examines synthetic strategies, therapeutic target patterns, and medicinal chemistry trends that shaped the 2025 approval landscape. By integrating regulatory context with mechanistic and synthetic insights, the review aims to provide a clearly positioned, analytically focused contribution to the growing literature of annual FDA approval summaries.

**Table 1:** Fluorinated drugs from novel drug approvals 2025

Drug name	Active ingredient	Approval date	FDA-approved use on approval date*	Recommended dosage by US FDA	Fluorinated unit
Journavx™	Suzetrigine	1/30/2025	To treat moderate to severe acute pain	50 mg orally every 12 hours	3,4-Difluoro-2-methoxyphenyl; CF_3_ on oxolane
Gomekli™	Mirdametinib	2/11/2025	To treat neurofibromatosis type 1 who have symptomatic plexiform neurofibromas not amenable to complete resection	2 mg m^−2^ orally twice daily (each 28 day cycle)	2,3,4-Trifluorobenzoyl; 2-fluoro-4-iodophenyl
Avmapki Fakzynja Co-Pack™	Avutometinib and defactinib	5/8/2025	For the treatment of KRAS-mutated recurrent low-grade serous ovarian cancer (LGSOC) after prior systemic therapy	AVMAPKI 3.2 mg administered orally twice weekly (Day 1 and day 4) for the first 3 weeks of each 4 week cycle FAKZYNJA 200 mg administered orally twice daily for the first 3 weeks of each 4 week cycle	3-Fluoropyridine; 5-(trifluoromethyl)pyrimidine
Ibtrozi™	Taletrectinib	6/11/2025	To treat locally advanced or metastatic ROS1-positive non-small cell lung cancer	600 mg orally once daily	3-Fluorobenzyl; imidazo[1,2-*b*]pyridazine
Zegfrovy™	Sunvozertinib	7/2/2025	To treat locally advanced or metastatic non-small cell lung cancer with epidermal growth factor receptor exon 20 insertion mutations, as detected by an FDA-approved test, with disease progression on or after platinum-based chemotherapyDrug trials snapshot	200 mg orally once daily with food	Trifluoromethyl (CF_3_)
Ekterly™	Sebetralstat	7/3/2025	To treat acute attacks of hereditary angioedemaDrug trials snapshot	300 mg orally as a single dose at attack onset	Multiple fluorines on aromatic rings
Wayrilz™	Rilzabrutinib	8/29/2025	To treat persistent or chronic immune thrombocytopenia that has not sufficiently responded to immunoglobulins, anti-D therapy, or corticosteroids	400 mg orally twice daily (total 800 mg day^−1^)	Fluorinated purine core
Inluriyo™	Imlunestrant	9/25/2025	To treat estrogen receptor-positive, human epidermal growth factor receptor 2-negative, estrogen receptor-1-mutated advanced or metastatic breast cancer with disease progression following at least one line of endocrine therapy	80 mg orally once daily	Multiple 4-fluorophenyl groups
Palsonify™	Paltusotine	9/25/2025	To treat acromegaly in adults who had an inadequate response to surgery and/or for whom surgery is not an option	40 mg orally once daily (adjustable range: 10–40 mg)	Trifluoromethyl (CF_3_)
Rhapsido™	Remibrutinib	9/30/2025	To treat chronic spontaneous urticaria in adults who remain symptomatic despite H1 antihistamine treatment	200 mg orally once daily	2,6-Difluorophenyl
Lynkuet™	Elinzanetant	10/24/2025	To treat moderate-to-severe vasomotor symptoms due to menopause	120 mg orally once daily in the morning	2,3-Difluorophenyl groups
Komzifti™	Ziftomenib	11/13/2025	To treat adults with relapsed or refractory acute myeloid leukemia with a susceptible nucleophosmin 1 mutation who have no satisfactory alternative treatment options	200 mg orally twice daily (total 400 mg day^−1^)	4-Fluorophenyl; difluorobenzyl
Nuzolvence™	Zoliflodacin	12/12/2025	To treat uncomplicated urogenital gonorrhea due to Neisseria gonorrhoeae	3000 mg (3 g) orally as a single dose	3-Fluorophenyl; difluoromethoxy
Nereus™	Tradipitant	12/30/2025	To prevent vomiting induced by motion in adults	A single oral dose of 85 mg or 170 mg	1,3-bis(trifluoromethyl)benzene

## 2 FDA-approved fluorine-containing small molecule drugs in 2025

### 2.1 Journavx™ (suzetrigine)

Journavx™ (suzetrigine, 1) is an orally administered sodium channel blocker developed by Vertex Pharmaceuticals Incorporated. It acts as a selective and potent blocker of NaV1.8, a voltage-gated sodium channel predominantly expressed in peripheral sensory neurons, including dorsal root ganglion neurons, where it plays a key role in pain signal transmission.^15^ It is indicated for the treatment of moderate to severe acute pain in adults and represents the first non-opioid analgesic with a novel mechanism of action approved in more than two decades. On January 30, 2025, the U.S. FDA approved Journavx™ 50 mg oral tablets for the treatment of acute pain in adults. The approval was supported by two Phase 3 randomized, double-blind, placebo- and active-controlled clinical studies, NCT055533669 (bunionectomy) and NCT0573134910 (abdominoplasty), conducted in 874 patients with moderate to severe acute postoperative pain.

The recommended dosage of Journavx™ is an initial 100 mg oral dose administered on an empty stomach, followed by 50 mg orally every 12 hours, starting 12 hours after the first dose, with or without food. The duration of therapy should be limited to the shortest period necessary to achieve the desired therapeutic effect; safety has not been established beyond 14 days of use.^16^ Adverse events occurring in ≥1% of patients and at a higher frequency than placebo include pruritus (2.1%), muscle spasms (1.3%), increased blood creatine phosphokinase (1.1%), and rash (1.1%). Compared with opioids, Journavx™ is associated with a lower incidence of nausea and vomiting and represents a significant advance in pain management by providing effective analgesia without the abuse potential, respiratory depression, or major gastrointestinal adverse effects typical of opioid therapy. Parallel use with strong CYP3A inhibitors must not be used; dose reduction is required with moderate CYP3A inhibitors or in patients with moderate hepatic impairment, and use is not permitted in cases of severe hepatic impairment.^16^

**Scheme 1:**
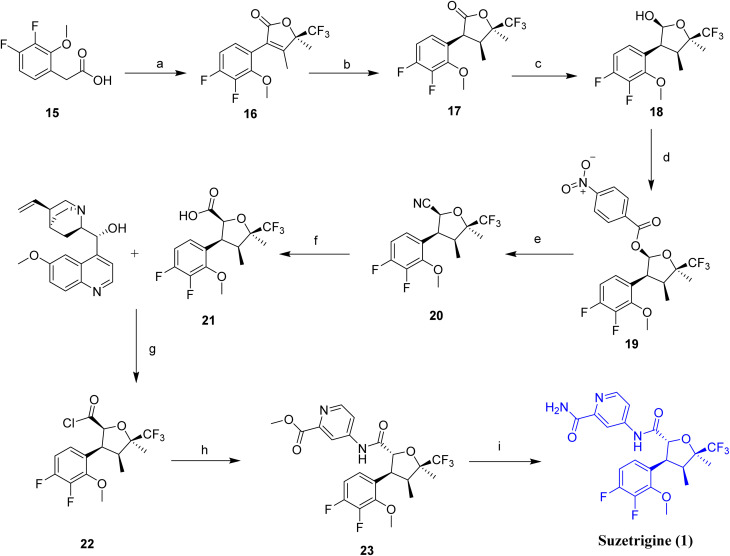
Synthesis of Suzetrigine (1). ^*a*^Reaction conditions: (a) (R)-4,4,4-trifluoro-3-hydroxy-3-methylbutan-2-one, 1,1′-carbonyldiimidazole, acetonitrile, 1.5 h at −2 to 0 °C, then for 5 h at 35 °C; (b) Pd/C, hydrogen/isopropyl alcohol for 30 h at 30 °C (c) diisobutylaluminum hydride (DIBAL-H), Toluene, 6.5 h at −31 to −26 °C; (d) 4-nitrobenzoyl chloride, TEA, Toluene at 0 °C; (e) trimethylsilanecarbonitrile, boron trifluoride diethyl etherate, toluene for 3 h at 20 °C; (f) quinine, isopropyl alcohol, *n*-heptane for 2 h at 65–70 °C; (g) hydrogen chloride/toluene, water at 20 °C, then for 4 h at 30 °C; (h) methyl 4-aminopicolinate, TEA, DCM for 4 h at 25 °C; (i) ammonia/methanol for 24 h at 20 °C.

The chemical synthesis of suzetrigine (1)^17^ was reported by Vertex Pharmaceuticals, which involves a multistep sequence, as outlined in Scheme 1. The synthetic route commences from (R)-4,4,4-trifluoro-3-hydroxy-3-methylbutan-2-one (15), which is activated with 1,1′-carbonyldiimidazole (CDI) to form the corresponding activated intermediate (16). Subsequent catalytic hydrogenation using Pd/C afforded the reduced intermediate (17), followed by selective reduction with diisobutylaluminium hydride (DIBAL-H) to yield compound 18. Esterification was achieved by acylation with 4-nitrobenzoyl chloride in the presence of triethylamine, providing compound (19). The nitrile group was then introduced using trimethylsilyl cyanide and boron trifluoride diethyl etherate, affording intermediate (20). Enantiomeric enrichment was carried out *via* quinine-mediated resolution (21), and subsequent acidic deprotection furnished the free intermediate (22). Coupling with methyl 4-aminopicolinate produced intermediate (23), which, upon aminolysis with ammonia, afforded the final product, suzetrigine (1).

### 2.2 Gomekli™ (mirdametinib)

Gomekli™ (mirdametinib, 2) is an orally bioavailable, selective, non-ATP-competitive inhibitor of mitogen-activated protein kinase kinases 1 and 2 (MEK1/2), exhibiting *K*_*i*_ values ≤1.1 nM against the activated MEK1 and MEK2 isoforms. It is being developed by SpringWorks Therapeutics, Inc., for the treatment of neurofibromatosis type 1 (NF1)-associated plexiform neurofibromas (PN), benign peripheral nerve sheath tumors that occur in approximately 40–50% of individuals with NF1 and are associated with significant morbidity, including pain, disfigurement, and neurological deficits. On February 11, 2025, the U.S. Food and Drug Administration (FDA) granted regular approval to Gomekli™ (mirdametinib, 2) for the treatment of adult and pediatric patients aged 2 years and older with neurofibromatosis type 1 who have symptomatic plexiform neurofibromas not amenable to complete surgical resection.^18^

Mirdametinib (2) is the first drug approved by the FDA for the treatment of neurofibromatosis type 1 in adults and children. This approval was based on a multicenter, single-arm trial (ReNeu; NCT03962543),^19^ that enrolled 114 subjects (58 adults, 56 pediatric). The trial reported a confirmed overall response rate of 41% (95% CI 29, 55) among adults and 52% (95% CI 38, 65) among pediatric subjects, with durable responses and improvements in disease-related symptoms, as assessed by BICR using volumetric MRI per the REiNS criteria. Serious adverse reactions include ocular toxicity (25%) and left ventricular dysfunction (20%).^19^ Monitoring for ocular adverse reactions, echocardiography, and embryo-fetal toxicity^20^ is indicated as a precaution for patients with NF1-associated symptomatic plexiform neurofibromas who are prescribed Mirdametinib (2).^19^

**Scheme 2:**
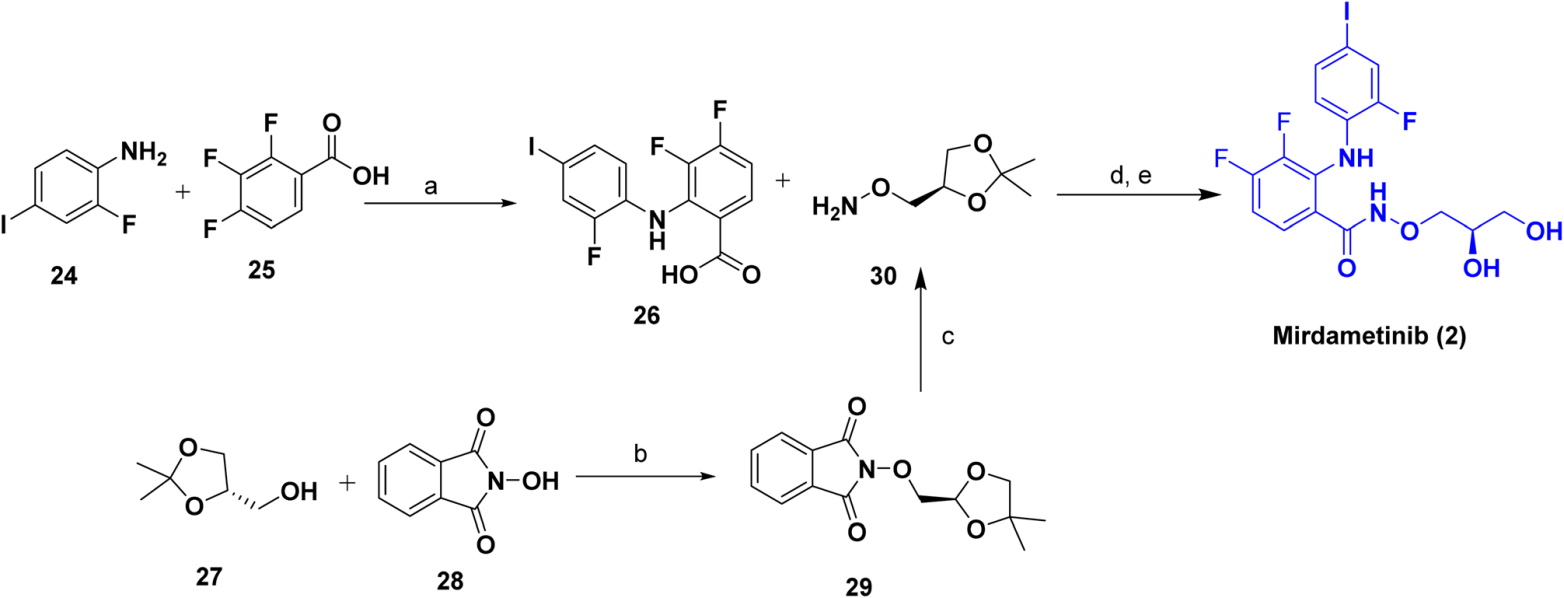
Synthesis of Mirdametinib (2). ^*a*^Reaction conditions: (a) LDA, THF, 0–5 °C, 1 h and rt, 3 h; (b) triphenylphosphine, Diethyl azodicarboxylate (DEAD), THF, 0 °C to rt, 18 h; (c) methylhydrazine, DCM, 3–5 °C, rt; (d) 4-methylmorpholine, diphenylphosphinic chloride, THF, rt, 18 h; (e) *p*-toluenesulfonic acid, methanol, H_2_O, rt, 18 h.

Scheme 2 outlines the synthesis of mirdametinib (2)^21^ that employs the biaryl amide and protected aminopropanediol intermediates. The starting reaction is an amide coupling reaction between 2-fluoro-4-iodoaniline (24) and 2,3,4-trifluorobenzoic acid (25) using lithium diisopropylamide (LDA). As a result, the biaryl amide 3,4-difluoro-2((2-fluoro-4-iodophenyl)amino)benzoic acid (26) is constructed, which possesses the difluorobenzamide core scaffold and iodo-substituted aniline moiety. Another precursor is a mitsunobu reaction between the alcohol (S)-(2,2-dimethyl-1,3-dioxolan-4-yl)methanol (27) and 2-hydroxy- isoindoline-1,3-dione (28) with Ph_3_P and diethyl azodicarboxylate (DEAD), providing the protected aminopropanediol (R)-O-((2,2-dimethyl-1,3-dioxolan-4yl)methyl)hydroxyl-amine (29) product. Conversion of the protected ester (29) to the corresponding hydroxylamine (30) was achieved by treatment with methylhydrazine. The final product was obtained *via* convergent amide coupling between intermediate 26 and the deprotected hydroxylamine 30, followed by acid-catalyzed hydrolysis to afford the corresponding vicinal diol, mirdametinib (2).

### 2.3 Avmapki Fakzynja Co-Pack™

Avmapki Fakzynja Co-PackTM (3), developed by Verastem, Inc., is a prescription combination therapy approved for the treatment of adult patients with recurrent low-grade serous ovarian cancer (LGSOC) harboring KRAS mutations who have received prior systemic therapy. LGSOC^12^ is a rare, chemoresistant ovarian cancer in which approximately 30% of tumors exhibit activating KRAS mutations that drive constitutive MAPK signaling.^22^ The Avmapki Fakzynja Co-Pack combines the MEK1 inhibitor avutometinib (3a) with the FAK inhibitor defactinib (3b) to achieve dual suppression of MAPK-dependent proliferation and adaptive resistance, providing a rational targeted strategy for KRAS-mutant LGSOC. The co-pack contains Avmapki (avutometinib, 3a) capsules and Fakzynja (defactinib, 3b) tablets packaged together to facilitate combination dosing and represents the first FDA-approved therapy specifically indicated for KRAS-mutant recurrent LGSOC. FDA accelerated approval was granted on May 8, 2025, based on efficacy results from the Phase 2 RAMP 201 clinical trial (NCT04625270); continued approval is contingent upon confirmation of clinical benefit in the ongoing Phase 3 RAMP 301 trial. The Co-Pack requires careful monitoring of eyes, skin, liver, and creatine kinase, with dose adjustment or interruption as needed, and should be avoided during pregnancy.^23,24^

**Scheme 3:**
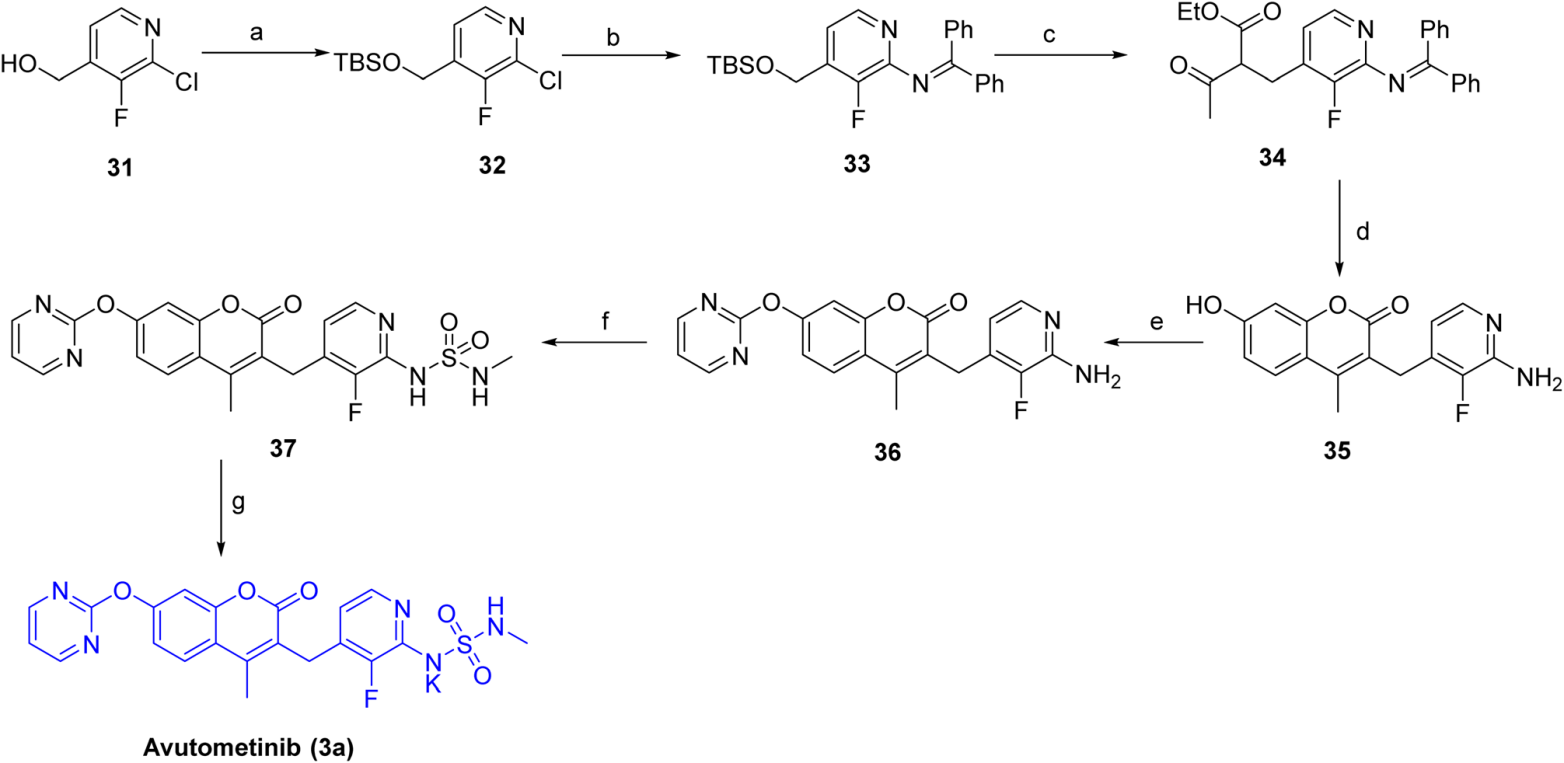
Synthesis of Avutometinib (3a). ^*a*^Reaction conditions: (a) *tert*-butyldimethylsilyl chloride, imidazole, DMF; (b) benzophenone imine, Pd_2_(dba)_3_, rac-BINAP, *t*-BuONa, toluene, 60 °C; (c) ethyl 3-oxobutanoate, *t*-BuOLi, NaI, THF, 50 °C, 3 h; (d) resorcinol, H_2_SO_4_; (e) 2-bromopyrimidine, NaH, DMF; (f) *N*-methylsulfamoyl chloride, pyridine, DMF; (g) KOH.

Scheme 3 outlines the synthesis of avutometinib (3a).^25^ The route begins with the protection of the hydroxy group in 31 using *tert*-butyldimethylsilyl chloride, affording the protected intermediate 32, followed by palladium-catalyzed C–N coupling with benzophenone imine to give 33. C–C bond formation with ethyl 3-oxobutanoate generated 34, which then underwent acid-mediated cyclization with resorcinol to furnish 35. The pyrimidine moiety was installed *via* nucleophilic substitution with 2-bromopyrimidine, producing 36, and the sulfonamide functionality was introduced by reaction with *N*-methylsulfamoyl chloride to give 37. Final deprotection and/or saponification using KOH afforded the target compound, avutometinib (3a).

**Scheme 4:**
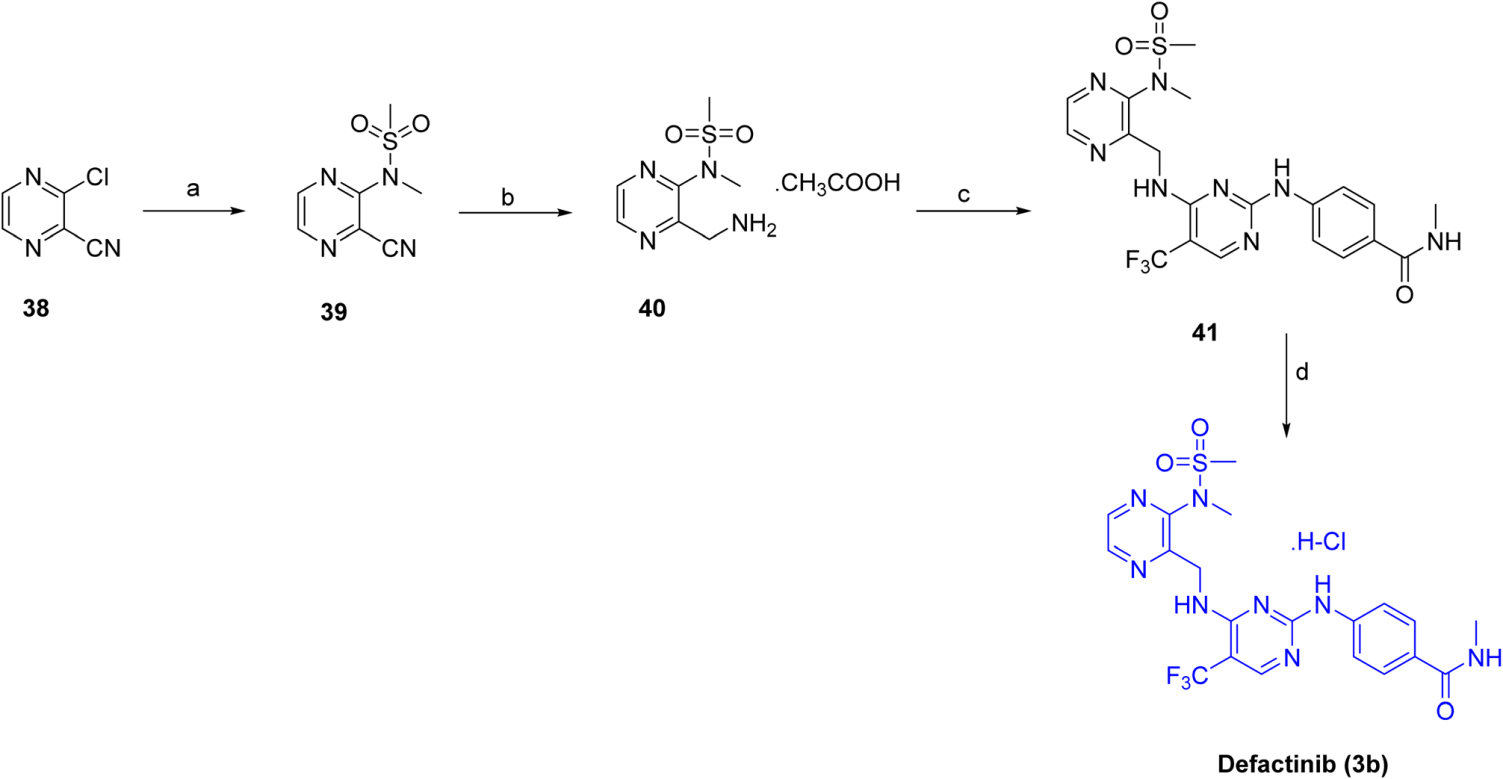
Synthesis of Defactinib (3b). ^*a*^Reaction conditions: (a) *N*-methyl-methanesulfonamide, Cs_2_CO_3_, acetonitrile, 80 °C, (b) H_2_, Pd/C in methanolic ammonia, AcOH, EtOAc, rt, 8 h; (c) 4-((4-chloro-5-(trifluoromethyl)pyrimidin-2-yl)amino)-*N*-methylbenzamide, DIEA, DCE/t-BuOH, 0 °C; (d) HCl in MeOH, 0 °C-rt, 2 h.

The target compound defactinib (3b)^26^ was synthesized as shown in Scheme 4. The synthesis began with the introduction of the *N*-methylsulfonamide group on 38 using *N*-methyl-methanesulfonamide in the presence of Cs_2_CO_3_, affording intermediate 39. The resulting intermediate 39 was then reduced with H_2_ and Pd/C to provide 40. Subsequent coupling of 40 with 4-((4-chloro-5-(trifluoromethyl)pyrimidin-2-yl)amino)-*N*-methylbenzamide in the presence of DIEA in DCE/*t*-BuOH installed the substituted pyrimidine amide functionality to obtain 41. Finally, treatment with HCl in MeOH resulted in deprotection, yielding the desired compound, defactinib (3b).

### 2.4 Ibtrozi™ (taletrectinib adipate)

Ibtrozi™ (taletrectinib, 4), a CNS-penetrant, orally bioavailable next-generation ROS1/Neurotrophic Tyrosine Receptor Kinase TK inhibitor, was approved by the FDA on June 11, 2025, and is being developed by Nuvation Bio Inc., for adults with locally advanced/metastatic ROS1-positive NSCLC. In TRUST-I/II (NCT04919811; *N* > 300),^27^ treatment-naive patients had an ORR of 85% (95% CI 77% to 91%) with a median DOR of 24.3 months and a median PFS of 23.9 months, and pretreated patients had an ORR of 50% and a median PFS of 9.4 months.^28^ Taletrectinib (4) also has strong CNS activity (CNS ORR 82%) and is active in patients with acquired ROS1 resistance mutations (G2032R, D2033N, L2086F). Compared with first-generation ROS1 inhibitors, Ibtrozi™ (taletrectinib, 4) has superior CNS penetration and broader resistance-mutation coverage.^29^

**Scheme 5:**
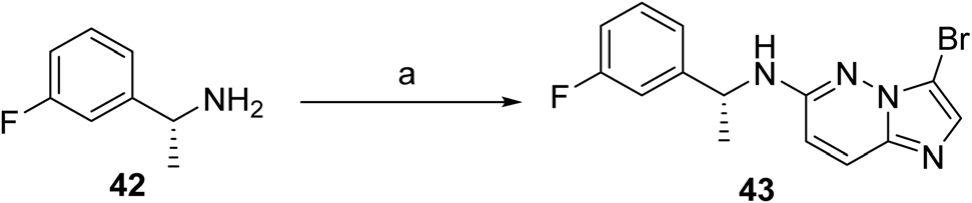
Synthesis of intermediate (43). ^*a*^Reaction conditions: (a) 3-bromo-6-chloroimidazo[1,2-*b*]pyridazine, KF, DMSO at 120 °C.

**Scheme 6:**
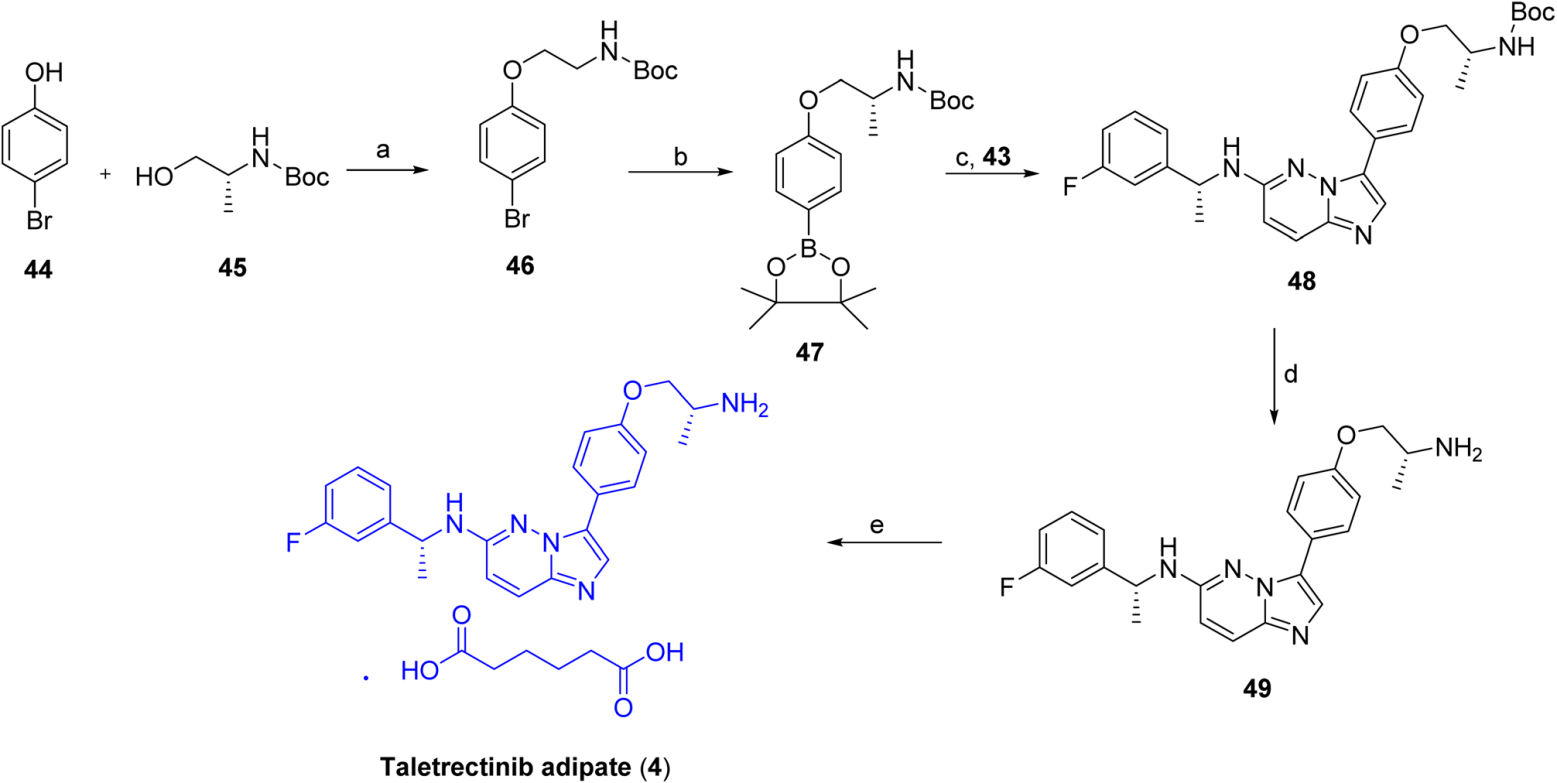
Synthesis of Taletrectinib (4). ^*a*^Reaction conditions: (a) *N*-Boc-*d*-alaninol, PPh_3_, DIAD, THF; (b) bis(pinacolato)diboron, PdCl_2_(dppf)·CH_2_Cl_2_, KOAc, 1,4-dioxane; (c) intermediate 43, PdCl_2_(dppf)·CH_2_Cl_2_, K_2_CO_3_, 1,4-dioxane/H_2_O; (d) HCl in MeOH/dioxane; (e) adipic acid in *n*-PrOH.

The synthetic route to taletrectinib (4)^30^ begins with a Mitsunobu reaction between *p*-bromophenol (44) and *N*-Boc-D-alaninol (45) employing PPh_3_ and DIAD in THF, providing *N*-Boc-1-(4-bromophenoxy)-2-(R)-propanamine (46) as depicted in Scheme 6. This intermediate undergoes Miyaura borylation with bis(pinacolato)diboron in the presence of PdCl_2_(dppf)·CH_2_Cl_2_ and KOAc in dioxane at 80 °C to generate the corresponding boronate ester (47). In a parallel pathway, nucleophilic aromatic substitution of 3-bromo-6-chloroimidazo[1,2-*b*]pyridazine with 1-(R)-(3-fluorophenyl)ethanamine (42), carried out in DMSO at 120 °C with KF, affords the secondary amine intermediate (43), as shown in Scheme 5. Subsequent Suzuki–Miyaura cross-coupling of this amine with boronate (47) using PdCl_2_(dppf)·CH_2_Cl_2_ and K_2_CO_3_ in refluxing dioxane/water furnishes *N*-Boc-protected taletrectinib (48). Final deprotection of the carbamate using HCl in methanol/dioxane yields the Taletrectinib free base (49), which is converted to taletrectinib adipate (4) by salt formation with adipic acid in *n*-propanol.

### 2.5 Ekterly™ (sunvozertinib)

Ekterly™ (sunvozertinib, 5) is an orally bioavailable, irreversible, selective EGFR TKI developed by Dizal (Jiangsu) Pharmaceutical Company Limited as the first-in-class targeted therapy for patients with the EGFR exon 20 insertion (exon20ins) mutation in NSCLC. On July 2, 2025, sunvozertinib (5) was approved by the US FDA *via* the accelerated approval pathway for use in adult patients with locally advanced or metastatic EGFR mutant (exon20ins) NSCLC whose disease has progressed on or after platinum-based chemotherapy. Sunvozertinib (5) is the only oral targeted NSCLC therapy approved for this historically difficult-to-drug EGFR mutation subtype of NSCLC. This mutation subtype occurs in 1–2% of NSCLC patients worldwide. Sunvozertinib (5) is a covalent, irreversible EGFR inhibitor. It selectively and irreversibly inhibited mutant EGFR with exon 20 insertion mutations, equivalent to wild-type EGFR, and 2- to 10-fold selectively inhibited phosphorylation and signaling of mutant *versus* wild-type EGFR, overcoming resistance to first- and second-generation EGFR TKIs, including exon 20 insertion mutations.^31^

**Scheme 7:**
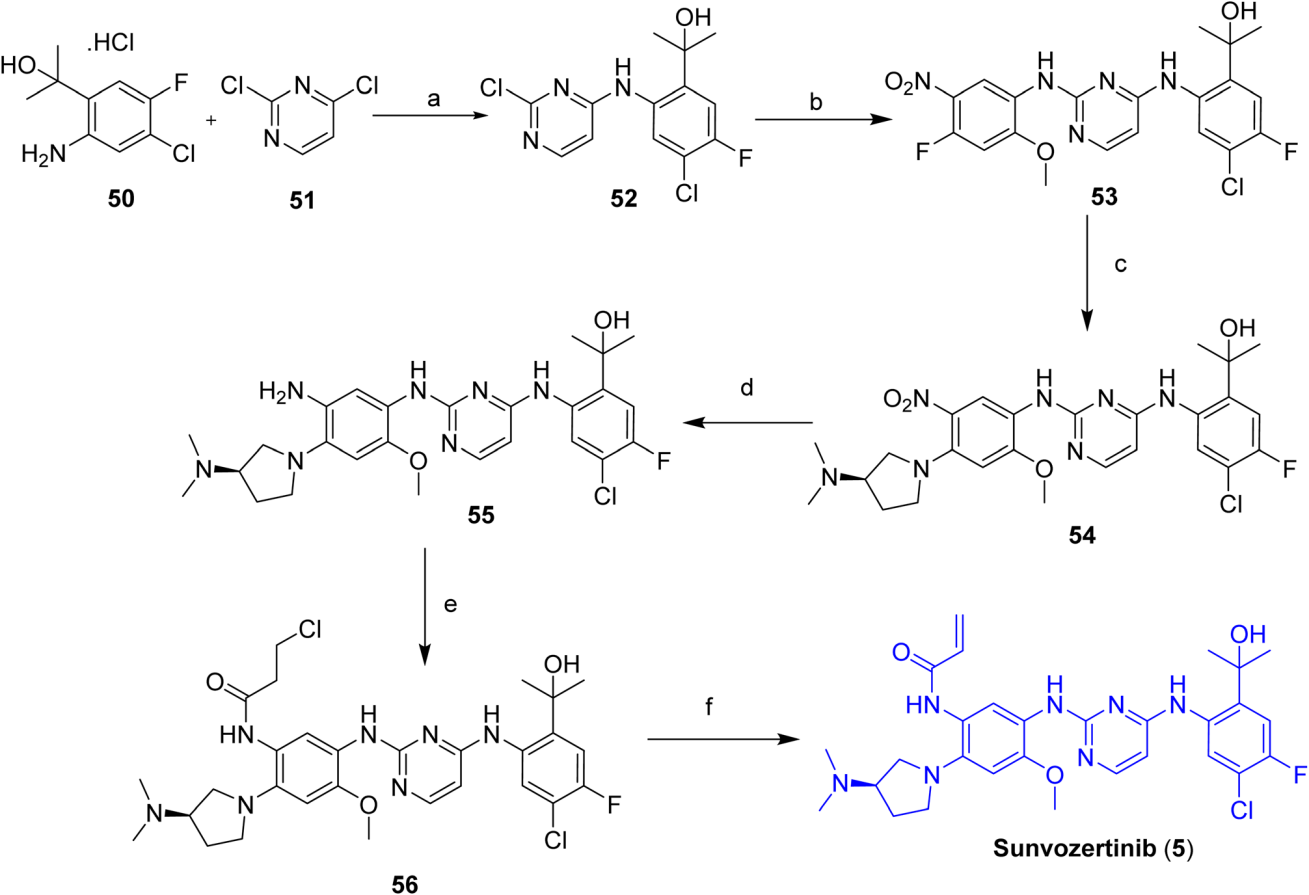
Synthesis of Sunvozertinib (5). ^*a*^Reaction conditions: (a) DIPEA, IPA; (b) 4-fluoro-2-methoxy-5-nitroaniline, IPA, TFA; (c) (R)-*N*,*N*-dimethylpyrrolidin-3-amine, DIPEA, K_2_CO_3_, ACN; (d) Pt/C, THF; (e) acyl chloride, THF, H_2_O; (f) NaOH, H_2_O, 20 °C, 2 h.

The synthetic route to sunvozertinib (5), shown in Scheme 7, begins with an alkaline-promoted nucleophilic substitution between compounds 50 and 51, yielding intermediate 52. This intermediate is subsequently reacted with 4-fluoro-2-methoxy-5-nitroaniline to form intermediate 53, which undergoes a further nucleophilic substitution with (*R*)-*N*,*N*-dimethylpyrrolidin-3-amine to afford intermediate 54. Catalytic hydrogenation of the nitro group in 54 using platinum on carbon provides the corresponding aniline derivative 55. Reaction of 55 with an acyl chloride furnishes amide 56, which, upon base-induced elimination, yields the target compound, sunvozertinib (5).^32^

### 2.6 Zegfrovy™ (sebetralstat)

Zegfrovy™ (sebetralstat, 6) is a novel, potent, and highly selective small-molecule plasma kallikrein inhibitor developed by KalVista Pharmaceuticals Inc. for the treatment of hereditary angioedema (HAE) in patients aged 12 years or older and represents the first orally administered on-demand therapy, approved by the US FDA on 3rd July 2025. The mechanism of action is competitive and reversible inhibition of plasma kallikrein, preventing the conversion of kininogen to kinins and thus the synthesis of bradykinin. This greatly reduces bradykinin, a major mediator of angioedema in HAE. In the randomized, double-blind, three-way, placebo-controlled phase 3 KONFIDENT trial (NCT05259917),^33^ sebetralstat (6) 600 mg showed a significantly shorter time to first symptom relief, a lower mean attack severity, greater attack resolution, and comparable safety to placebo; headache was the most commonly reported adverse event. These results support the use of sebetralstat (6) as a self-administered oral therapy for treating patients with acute HAE attacks at unmet need.^33^

**Scheme 8:**
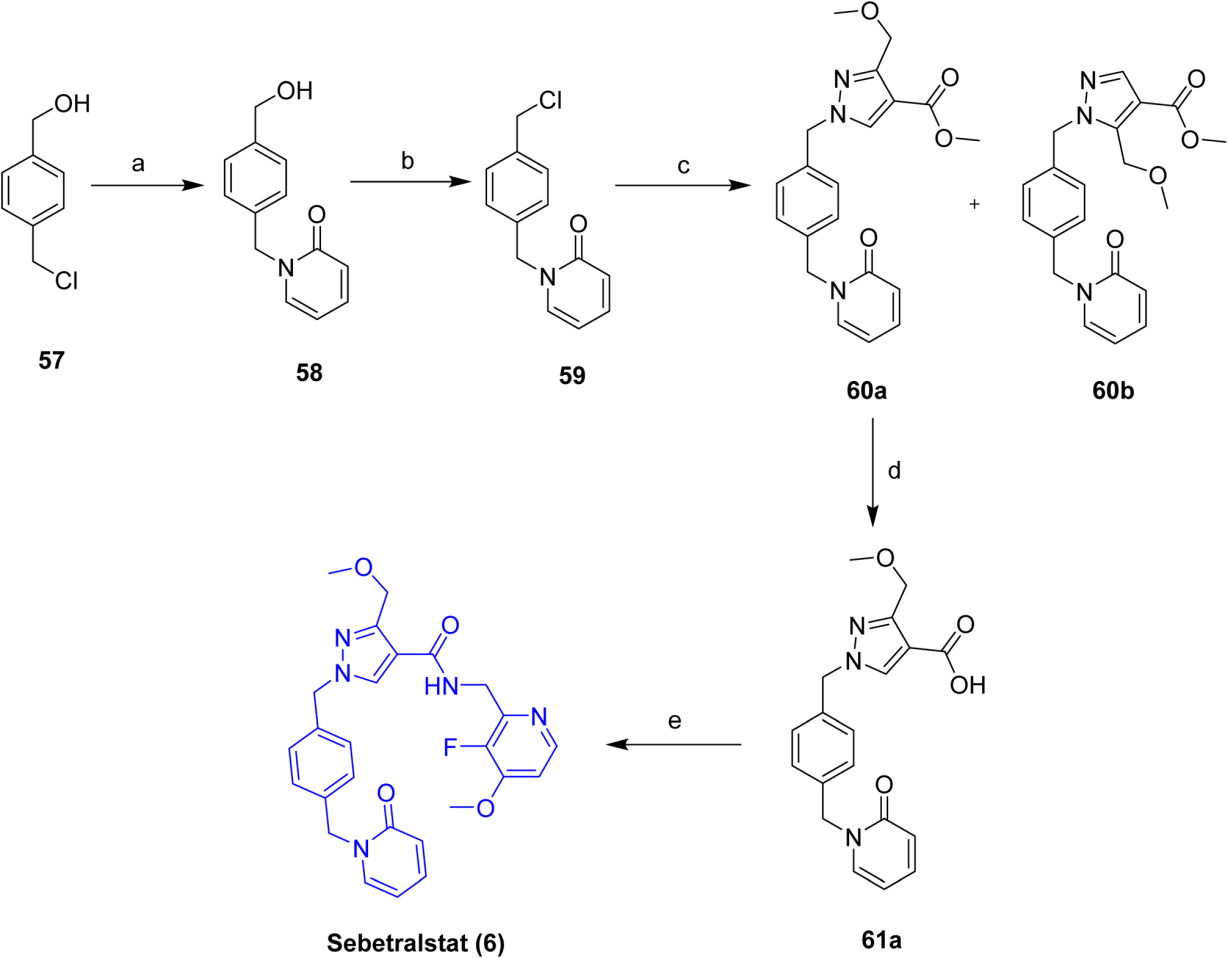
Synthesis of Sebetralstat (6). ^*a*^Reaction conditions: (a) 2-hydroxypyridine, K_2_CO_3_, acetone, 50 °C, 18 h, 78%; (b) methanesulfonyl chloride, TEA, dichloromethane, rt, 18h, 93%; (c) methyl 3-(methoxymethyl)-1*H*-pyrazole-4-carboxylate, K_2_CO_3_, DMF, 60 °C, 18 h, 54%; (d) 60a, NaOH, THF-MeOH-H_2_O, rt, 18 h, 34%; (e) C-(3-fluoro-4-methoxy-pyridin-2-yl)-methylamine, HATU, TEA, dichloromethane, rt, 4 h, 64%.

The preparation of sebetralstat (6)^34^ follows a 5-step linear assembly of classical organic reactions to produce the pyrazole series of plasma kallikrein inhibitors, Scheme 8. This process begins with regioselective *N*-alkylation of 2-hydroxypyridine with 4-(chloromethyl)benzyl alcohol 57 using K_2_CO_3_ in acetone at 50 °C, producing the *N*-benzyl pyridone alcohol intermediate 58 in 78% yield. This intermediate is mesylated to give the corresponding chloride with methanesulfonyl chloride and Et_3_N in dichloromethane to obtain the benzyl chloride intermediate 59 in 93% yield. The *N*-alkylation of 59 with methyl 3-(methoxymethyl)-1*H*-pyrazole-4-carboxylate in the presence of K_2_CO_3_ in DMF at 60 °C proceeds to a mixture of regioisomeric products, with the N-1 alkylated pyrazole methyl ester 60a (54% yield) favored over the N-2 regioisomer 60b; these can be separated using column chromatography techniques. Base-mediated ester hydrolysis of methyl ester 60a with NaOH in a THF-MeOH-H_2_O mixture at r.t. gives carboxylic acid intermediate 61a (34% yield). Final 5-HT1A agonists Sebetralstat (6) are assembled by amide coupling of 61a with C-(3-fluoro-4-methoxypyridin-2-yl)methylamine using HATU/TEA in dichloromethane at r.t. to produce 64% yield.

### 2.7 Wayrilz™ (rilzabrutinib)

Wayrilz™ (rilzabrutinib, 7) is developed by Sanofi for the treatment of immune thrombocytopenia (ITP) and is a reversible covalent inhibitor of Bruton's tyrosine kinase (BTK). The U.S. FDA approved rilzabrutinib (7) on August 29, 2025, for the treatment of adult patients 18 years of age or older with persistent or chronic ITP who have had an insufficient response to other medical therapies. The drug exhibits a rapid on-rate and a slow off-rate for BTK binding. *In vivo*, more than 80% BTK occupancy was achieved within 1 h and was maintained for more than 24 h. Rilzabrutinib (7) inhibits multiple steps in disease pathogenesis, including B-cell activation and differentiation, production of pathogenic autoantibodies, and Fcγ receptor-mediated phagocytosis of platelets by splenic and hepatic macrophages. In the Phase 3 LUNA3 trial (NCT04562766),^35^ the proportion of patients achieving a durable platelet response (platelet count ≥50 × 10^9^/L for at least two-thirds of an ≥8 week period during the final 12 weeks of treatment) was 23.3% in patients receiving Wayrilz™ (rilzabrutinib, 7) 400 mg b.i.d., compared with none in the placebo group (*P* < 0.0001). The median time to a sustained platelet response with Wayrilz™ (rilzabrutinib, 7) was 15 days, and a 52% reduction in rescue-therapy use was observed with the 100 mg and 200 mg doses, along with improvements in bleeding and physical fatigue. Gastrointestinal adverse events were the most common treatment-emergent adverse events (AEs) and were predominantly mild to moderate in severity (32% diarrhea; 20% nausea), with treatment discontinuation due to GI AEs reported in 4.5% of patients. Potential clinical limitations include treatment-emergent adverse events, drug–drug interactions with CYP3A inhibitors and inducers, and with gastric-secretion-reducing agents, moderate to severe hepatic or renal impairment, and the need to further establish safety, efficacy, and appropriate prior lines of therapy in pediatric populations.^36,37^

**Scheme 9:**
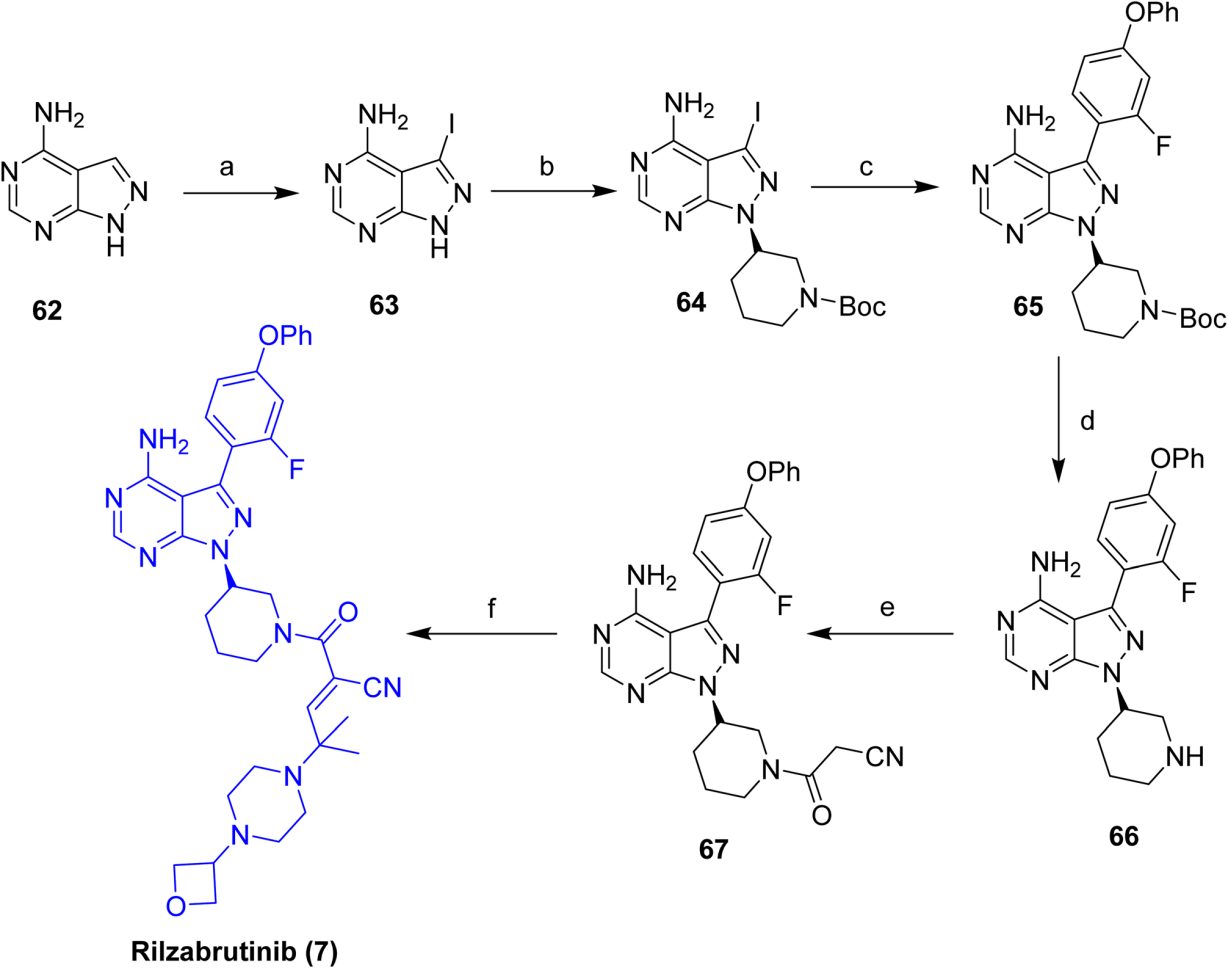
Synthesis of Rilzabrutinib (7). ^*a*^Reaction conditions: (a) *N*-iodosuccinamide, DMF, 80 °C; (b) *tert*-butyl (*S*)-3-hydroxypiperidine-1-carboxylate, PPh_3_, DIAD, THF; (c) (2-fluoro-4-phenoxyphenyl)boronic acid, K_2_CO_3_, 80 °C, dioxane/water; (d) TFA, CH_2_Cl_2_; (e) cyanoacetic acid, HOBt, EDCI, CH_2_Cl_2_; (f) 2-methyl-2-(4-(oxetan-3-yl)piperazin-1-yl)propanal, pyrrolidine, TMSCl, CH_2_Cl_2_.

In 2022, Sanofi disclosed a synthetic route to rilzabrutinib (7, Scheme 9).^38^ The synthesis starts from 4-aminopyrazolo[3,4-*d*]pyrimidine (62), which is converted into the cyanoacetamide intermediate 67 through a five-step sequence comprising iodination (63), a Mitsunobu reaction (64), Suzuki–Miyaura cross-coupling (65), Boc deprotection (66), and final amidation with cyanoacetic acid, delivering 67 in an overall yield of 3%. The terminal step involves a Knoevenagel condensation of 67 with 2-methyl-2-(4-(oxetan-3-yl)piperazin-1-yl)propanal, producing a 9 : 1 E/Z mixture, from which the Z-isomer was removed by Chiralpak IC HPLC purification to afford Rilzabrutinib (7). Despite its feasibility, this route suffers from low cumulative efficiency (0.5% overall yield across eight steps), elevated costs, and operational complexity, underscoring the need for improved, more economical synthetic strategies. More recently, an alternative synthesis of rilzabrutinib (7) has been reported that proceeds *via* an E-configured cyanoacrylic acid intermediate (Scheme 9).

### 2.8 Inluriyo™ (imlunestrant)

Inluriyo™ (imlunestrant, 8) is an orally bioavailable, next-generation selective estrogen receptor degrader (SERD) developed by Eli Lilly and Company for the treatment of estrogen receptor positive (ER+), HER2-negative, and ESR1-mutant advanced or metastatic breast cancer in patients whose disease has progressed following one or more lines of endocrine therapy. It's an oral SERD that induces conformational changes and subsequent proteolytic degradation of the ER, as well as inhibition of transcriptional activity mediated by the ER in ER-positive breast cancer, including cases with activating ESR1 mutations that are resistant to aromatase inhibitors and other agents. On September 25, 2025, the U.S. Food and Drug Administration (FDA) approved Inluriyo™ (imlunestrant, 8) based on clinical trial evidence of antitumor activity in endocrine-resistant, molecularly defined patient populations. Imlunestrant (8), a selective mutation-relevant endocrine therapy targeting a major mechanism of resistance in metastatic ER+ breast cancer, demonstrated an acceptable safety profile consistent with selective estrogen receptor degradation.^39^

**Scheme 10:**
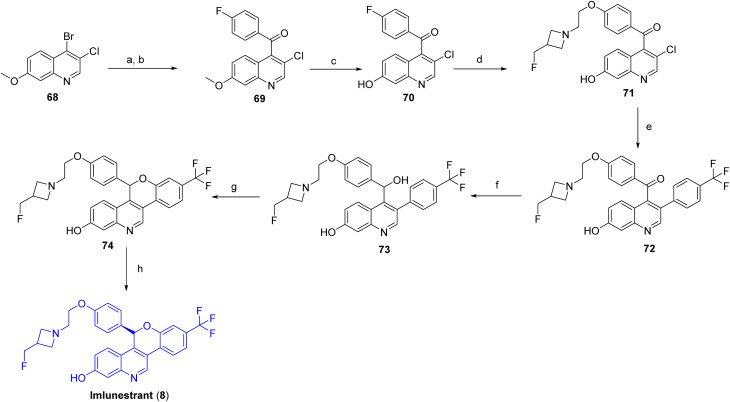
Synthesis of Imlunestrant (8). ^*a*^Reaction conditions: (a) *i*-PrMgCl, THF; (b) 4-fluorobenzoyl chloride in THF; (c) BBr_3_, CH_2_Cl_2_; (d) 2-(3-(fluoromethyl)azetidin-1-yl)ethan-1-ol, NaH, DMF; (e) (4-(trifluoromethyl)phenyl)boronic acid, XPhos-Pd-G2 and K_2_CO_3_, 2-methyl-2-butanol/H_2_O; (f) LiBHEt3 in THF/dioxane; (g) NaH, THF; (h) chiral SFC.

Scheme 10 outlines the synthetic route to imlunestrant (8).^40^ The synthesis begins with 4-bromo-3-chloro-7-methoxyquinoline (68), which undergoes metal–halogen exchange with *i*-PrMgCl, followed by Grignard acylation with 4-fluorobenzoyl chloride to afford (3-chloro-7-methoxy-4-quinolyl)(4-fluorophenyl)methanone (69). Selective demethylation of the 7-methoxy substituent with BBr_3_ furnishes the corresponding 7-hydroxyquinoline intermediate (70), which is subjected to NaH promoted alkylation with 2-[3-(fluoromethyl)azetidin-1-yl]ethanol to provide the ether intermediate (71). Subsequent Suzuki–Miyaura cross-coupling of 71 with [4-(trifluoromethyl)phenyl]boronic acid yields diaryl ketone (72), which is reduced using LiBHEt_3_ to generate secondary alcohol (73). Base-induced intramolecular cyclization of this alcohol produce the racemic chromeno[4,3-*c*]quinolin-2-ol framework (74), and final resolution by chiral supercritical fluid chromatography (SFC) affords enantiomerically pure imlunestrant (8).

### 2.9 Palsonify™ (paltusotine)

Palsonify™ (paltusotine, 9) is an orally active, nonpeptide agonist of the somatostatin receptor type 2 (SSTR2) and a selective oral somatostatin analog approved for the treatment of acromegaly in adults who have had an inadequate response to surgery or for whom surgery is not an option. It received FDA approval on September 25, 2025. Paltusotine (9) is developed by Crinetics Pharmaceuticals, Inc., and acts as an agonist of the SSTR2 receptor, thereby reducing excessive growth hormone production. The predictable absorption and elimination characteristics of paltusotine (9), together with a favorable metabolic profile that does not generate disproportionate or highly reactive metabolites, support the safety of long-term treatment. Paltusotine (9) offers a targeted, medically supervised therapeutic option for patients requiring clinical control of growth hormone who are not candidates for, or unlikely to benefit from, surgical intervention.^41^

**Scheme 11:**
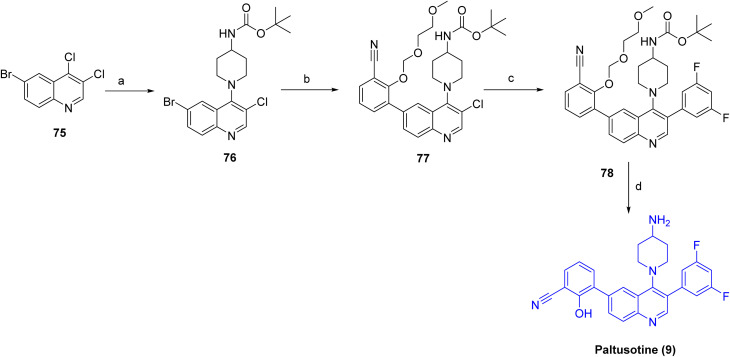
Synthesis of Paltusotine (9). ^*a*^Reaction conditions: (a) *tert*-butyl piperidin-4-ylcarbamate, DIPEA, 60 °C, 63% (b) 2-(2-methoxy-ethoxymethoxy)-3-(4,4,5,5-tetramethyl-[1,3,2]dioxaborolan-2-yl)-benzonitrile, PdCl_2_dppf, KOAc, 80 °C, 1 h; (c) 3, 5-difluorophenyl boronic acid, Pd (amphos)Cl_2_, K_2_CO_3_, 95 °C; (d) TFA, ACN/water.

As shown in Scheme 11, paltusotine (9)^42^ was prepared *via* a modular, stepwise cross-coupling approach. Initial nucleophilic aromatic substitution of 6-bromo-3,4-dichloroquinoline (75) with *tert*-butyl piperidin-4-ylcarbamate afforded the quinoline-piperidine intermediate (76). This intermediate was subjected to Suzuki–Miyaura cross-coupling with a protected 2-(2-methoxyethoxymethoxy)-3-boronate benzonitrile to yield arylated species 77, which was further subjected to a second Suzuki coupling with 3,5-difluorophenylboronic acid to provide the fully substituted quinoline derivative 78. Final acidic removal of the *tert*-butyl carbamate, followed by conversion to the hydrochloride salt, furnished the target compound paltusotine (9) in high purity.

### 2.10 Rhapsido™ (remibrutinib)

Rhapsido™ (remibrutinib, 10) is a highly selective, orally bioavailable small-molecule inhibitor of Bruton's tyrosine kinase (BTK) developed by Novartis for disorders driven by excessive immune activation. Remibrutinib (10) irreversibly inhibits BTK, thereby blocking FcεRI-mediated activation of mast cells and basophils and reducing the downstream release of pro-inflammatory mediators in patients with chronic spontaneous urticaria (CSU) who have not responded to maximal H1 antihistamine therapy. On September 30, 2025, Rhapsido™ (remibrutinib, 10) received approval from the U.S. Food and Drug Administration for the treatment of adults with CSU who continue to exhibit active disease despite H1 antihistamine use. Clinical studies demonstrated statistically significant reductions in urticaria activity and improvements in disease-related quality of life, with tolerability consistent with selective BTK inhibition in this population with substantial unmet medical need. As the first BTK inhibitor approved for CSU, remibrutinib (10) offers a mechanism-based, targeted therapeutic option with a manageable safety profile. However, monitoring for treatment-emergent adverse events associated with kinase inhibition remains warranted. At present, its approved use is limited to chronic spontaneous urticaria and does not extend to other forms of urticaria. Compared with non-specific immunosuppressive therapies, remibrutinib (10) may offer improved clinical utility and safety.^43^

**Scheme 12:**
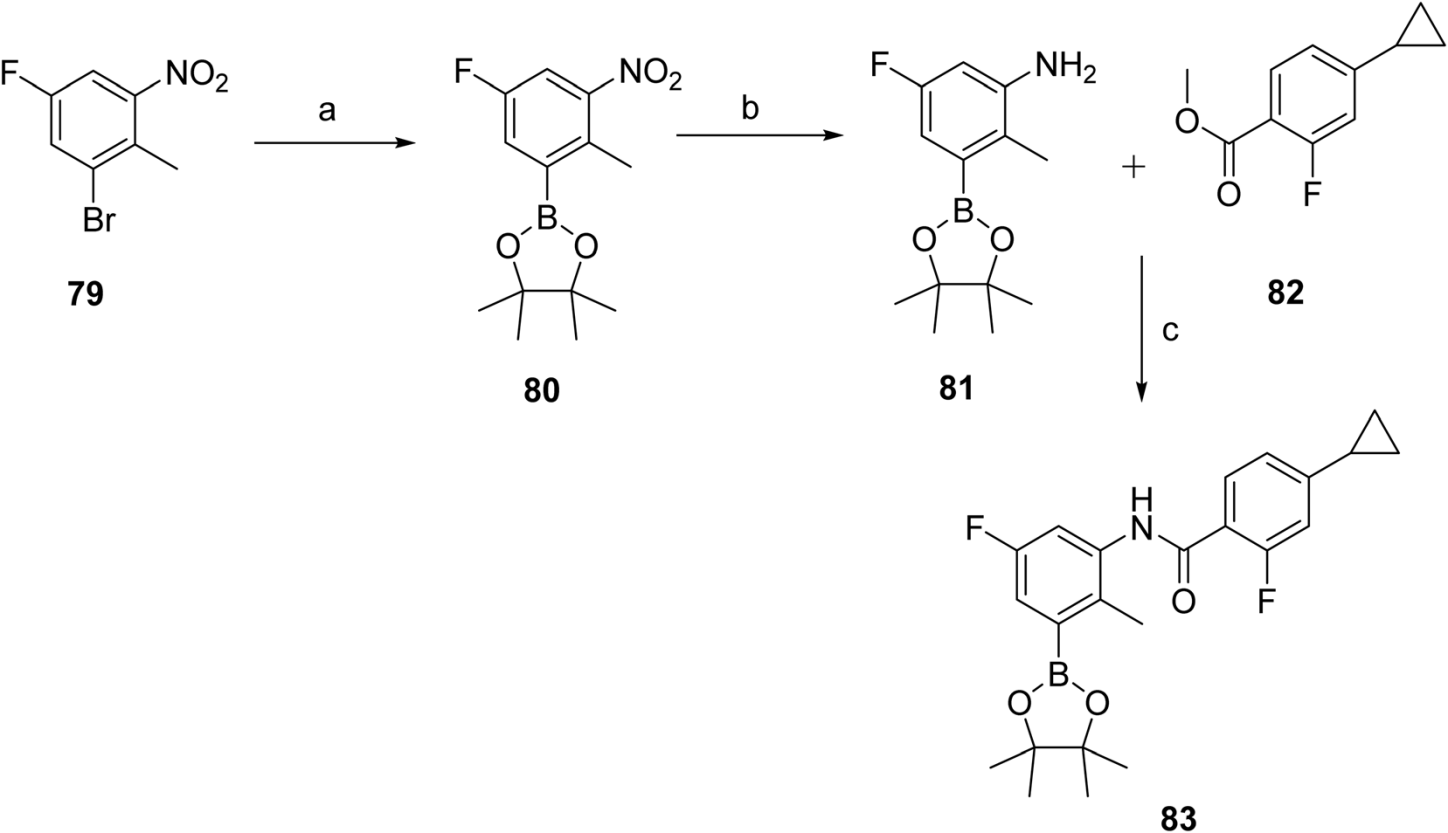
Synthesis of intermediate (83). ^*a*^Reaction conditions: (a) BISPIN, Pd(dppf)Cl_2_·DCM, KOAc, dioxane, 100 °C, 3.5 h, 92%; (b) H_2_, Pd/C, MeOH, RT, 7 h, 93%; (c) NaHMDS (1 M in THF), THF, RT, 4 h, 76%.

**Scheme 13:**
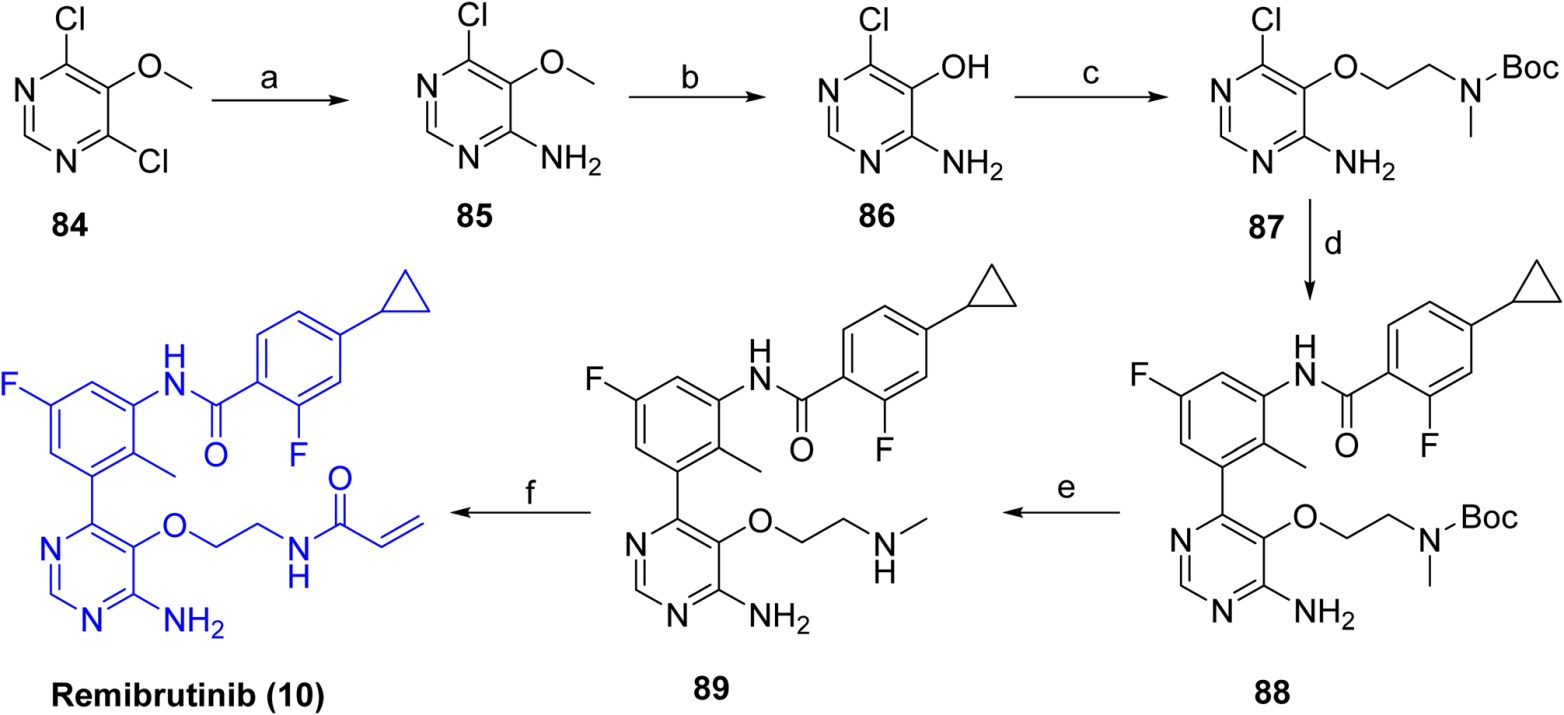
Synthesis of Remibrutinib (10). ^*a*^Reaction conditions: (a) NH_4_OH, 2-propanol, 70 °C, 48 h, 94%; (b) BBr_3_, DCM, 40 °C, 3 h, 59%; (c) *N*-Boc-*N*-methyl-2-hydroxyethylamine, DIAD, Smopex-301, THF, 60 °C, 2 h, 53%; (d) 83, PdCl_2_(PPh_3_)_2_, aq Na_2_CO_3_, DME, water, microwave, 110 °C, 25 min, 74%; (e) TFA, DCM, rt, 12 h; (f) acrylic acid, DIPEA, T3P (50% in DMF), DMF, rt, 2 h, 45% over 2 steps.

The synthetic route to remibrutinib (10)^44^ is presented in Schemes 12 and 13. Scheme 12 outlines the construction of the upper fragment of the molecule, corresponding to intermediate 83. In this sequence, 1-bromo-5-fluoro-2-methyl-3-nitrobenzene (79) underwent Miyaura borylation with bis(pinacolato)diboron using Pd(dppf)Cl_2_·DCM and potassium acetate to afford boronic ester 80, which was then reduced by catalytic hydrogenation to give aniline 81. Boronic ester 83 was prepared *via* NaHMDS-mediated amide formation between aniline 81 and cyclopropyl intermediate 82.

Scheme 13 depicts the completion of the synthesis of Remibrutinib (10), which commenced with the construction of the lower fragment from 2,4-dichloro-3-methoxypyrimidine (84), which was treated with ammonia under pressure, affording the corresponding aminopyrimidine 85. Subsequent demethylation with boron tribromide afforded aminopyrimidinol 86. Introduction of the linker unit was achieved *via* a Mitsunobu reaction with *N*-Boc-*N*-methyl-2-hydroxyethylamine employing DIAD and Smopex-301, a polymer-supported triphenylphosphine reagent, to give intermediate 87. Suzuki–Miyaura coupling of 87 with intermediate 83 furnished Boc-protected intermediate 88. Final Boc deprotection to 89, followed by amide coupling with acrylic acid using propylphosphonic anhydride (T3P), delivered Remibrutinib (10).

### 2.11 Lynkuet™ (elinzanetant)

Lynkuet™ (elinzanetant, 11), developed by Bayer Health Care Pharmaceuticals, Inc., is an orally administered, non-hormonal, selective dual antagonist of neurokinin-1 (NK-1) and neurokinin-3 (NK-3) receptors. It received approval from the U.S. Food and Drug Administration on October 24, 2025, for the treatment of moderate to severe vasomotor symptoms (VMS), including hot flashes and night sweats, becoming the third FDA-approved nonhormonal therapy for menopause-associated VMS. These symptoms are associated with dysregulation of peptide neurotransmitters and neuropeptides, including neurokinin A, substance P, neurokinin B (NKB), and kisspeptin, which play key roles in neuroendocrine and reproductive signaling. During menopause, reduced estrogen levels result in hyperactivation of kisspeptin/NKB/dynorphin (KNDy) neurons, leading to impaired hypothalamic thermoregulation and the onset of VMS. In clinical studies, elinzanetant (11) also demonstrated dose-dependent reductions in serum estradiol and luteal-phase progesterone levels. A limitation of elinzanetant (11) is that its approved indication is restricted to menopausal vasomotor symptoms, and long-term real-world safety data remain limited.^45–48^

**Scheme 14:**
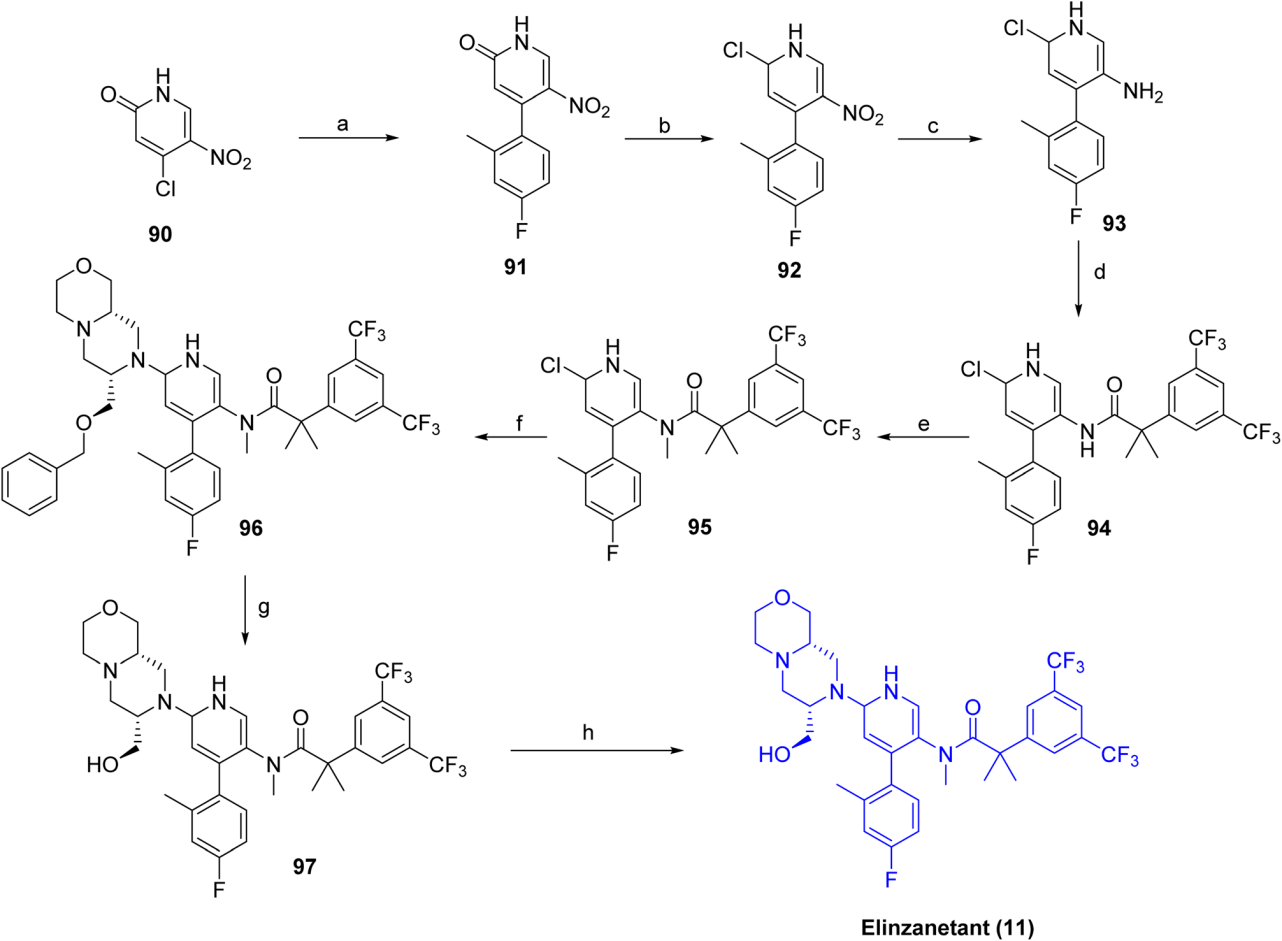
Synthesis of Elinzanetant (11). ^*a*^Reaction conditions: (a) (4-fluoro-2-methylphenyl)boronic acid, (Pd(PPh_3_)_4_), K_2_CO_3_, 1,4 dioxane; (b) POCl_3_, DMF; (c) Pt/C, EtOAc, rt, 2 h; (d) 2-(3,5-bis(trifluoromethyl)phenyl)-2-methylpropanoic acid, DCM, oxalyl chloride; (e) CsCO_3_, CH_3_Cl, DMF, (f) bis(tri-*tert*-butylphosphine)palladium, NaOtBu; (g) H_2_, Pd/C, isopropanol (IPA), HCl, IPA, (h) NaOH, MTBE, IPA.

As shown in Scheme 14,^49^ the synthesis of elinzanetant (11) begins with a palladium-catalyzed cross-coupling of starting material 90 with an arylboronic acid under basic conditions in 1,4-dioxane to afford the coupled product 91. Formylation of 91 with POCl_3_/DMF afforded the corresponding chloro derivative 92, which was then reduced over Pt/C in ethyl acetate to give the amine intermediate 93. Coupling of amine 93 with the appropriate carboxylic acid produced amide 94. Subsequent base-mediated methylation in DMF introduced the required methyl substituent to afford 95, followed by palladium-catalyzed coupling with an oxazine derivative to deliver intermediate 96. Final hydrogenation over Pd/C in isopropanol under acidic conditions effected the selective hydrogenolytic removal of the benzyl ether protecting group to give 97, and purification by basic workup with NaOH in MTBE/IPA afforded elinzanetant (11).

### 2.12 Komzifti™ (ziftomenib)

Komzifti™ (ziftomenib, 12) is an orally bioavailable, potent small-molecule inhibitor of the menin-KMT2A (MLL) protein–protein interaction, developed by Kura Oncology, Inc., as a targeted epigenetic therapy for acute myeloid leukemia (AML). Ziftomenib (12) selectively disrupts menin-KMT2A binding, suppressing leukemogenic transcriptional programs in AML characterized by nucleophosmin-1 (NPM1) mutations and KMT2A rearrangements. On November 13, 2025, the U.S. FDA granted accelerated approval to ziftomenib (12) for the treatment of adult patients with relapsed or refractory AML harboring a susceptible NPM1 mutation and lacking satisfactory alternative therapeutic options. This approval was supported by evidence of clinically meaningful anti-leukemic activity and durable responses in heavily pretreated patient populations. As the first epigenetic therapy selectively active in genetically defined AML subsets with substantial unmet medical need, ziftomenib (12) represents a significant advance; however, its clinical use may be constrained by treatment-emergent adverse events commonly associated with intensive targeted therapies, necessitating careful patient monitoring and potential dose modification.^50^

**Scheme 15:**
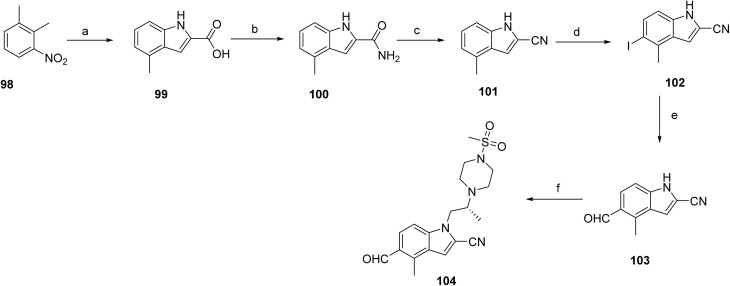
Synthesis of intermediate (104). ^*a*^Reaction conditions: (a) NaH, THF, diethyloxalate, tert-amyl alcohol; (b) oxalyl chloride, DCM; (c) POCl_3_, toluene; (d) DCM, BF_3_.OEt_2_, NIS; (e) P(Cy)7-HBF7, Na_2_CO_3_, DMF, Pd(OAc)_2_, Et3SiH; (f) Tf_2_O, DIPEA, (*R*)-2-(4-(methylsulfonyl)piperazin-1-yl)propan-1-ol, Cs_2_CO_3._

The synthesis of ziftomenib (12)^51^ involves the independent preparation of key intermediates 104 and 111, with the route to intermediate 104 outlined in Scheme 15. Compound 99 was obtained by base-promoted condensation of starting material 98 with diethyl oxalate in *tert*-amyl alcohol. Subsequent activation of 99, followed by amidation, afforded intermediate 100, which was converted to intermediate 101 upon treatment with phosphorus oxychloride. Electrophilic iodination of 101 with *N*-iodosuccinimide in the presence of boron trifluoride etherate afforded iodinated intermediate 102. This intermediate was subjected to a palladium-catalyzed carbonylative coupling to generate compound 103. In the final sequence, the hydroxyl group of 103 was converted to the corresponding triflate, which underwent base-mediated coupling with (R)-2-(4-(methylsulfonyl)piperazin-1-yl)propan-1-ol to provide intermediate 104.

**Scheme 16:**
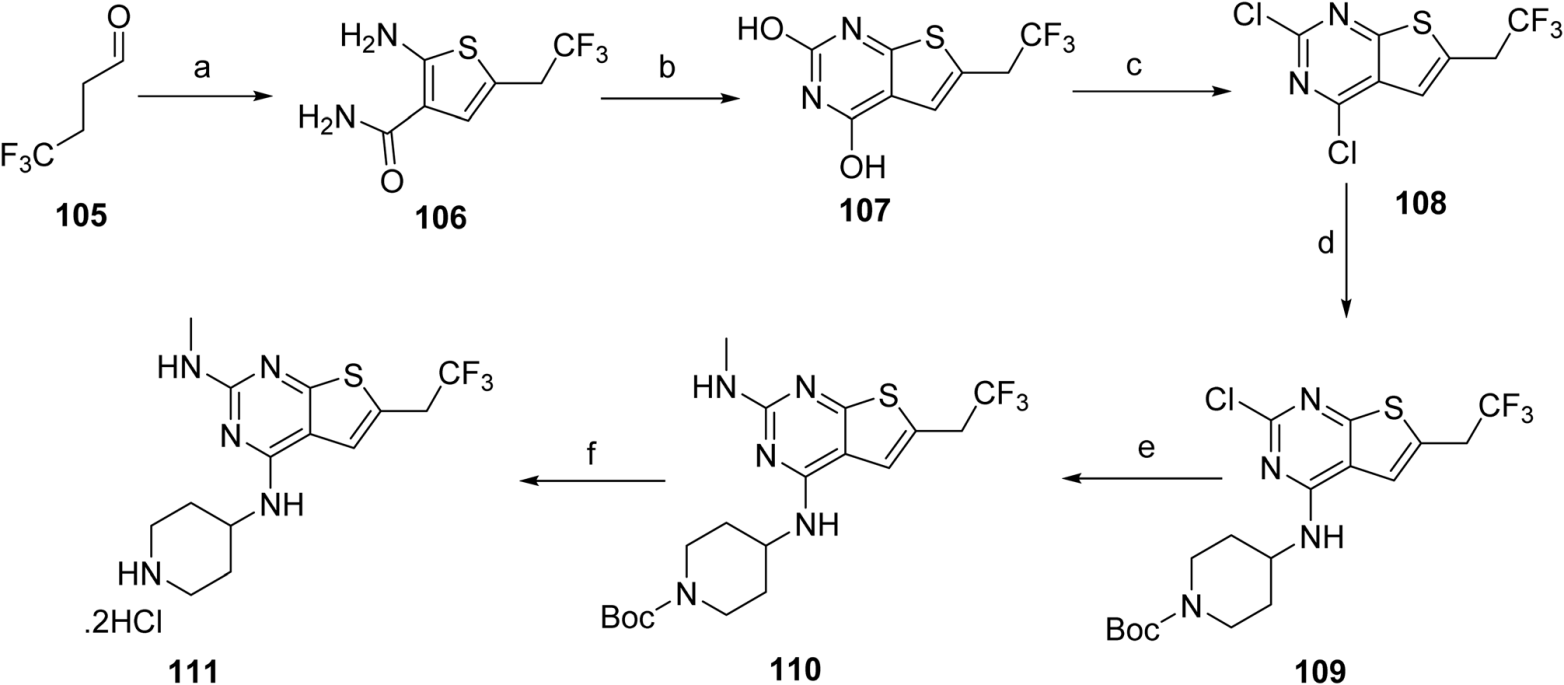
Synthesis of intermediate (111). ^*a*^Reaction conditions: (a) cyanoacetamide, Me-THF, DMF, S_8_, TEA; (b) CDI, Me-THF; (c) tetraethyl ammonium chloride, POCl_3_; (d) DIPEA, *tert*-butyl 4-aminopiperidine-1-carboxylate; (e) TEA, MeNH_2_, EtOH, H_2_O; (f) 4 M HCl, MeOH.

The synthesis of intermediate 111, shown in Scheme 16, begins with an aldehyde (105) that undergoes a base-mediated multicomponent thiophene annulation with 2-cyanoacetamide and elemental sulfur to afford the substituted aminothiophene carboxamide 106. Cyclization of 106 using a carbonyl-activating reagent affords the thieno[2,3-*d*]pyrimidine-2,4-diol scaffold (107). Activation of the heterocycle by chlorination yields the dichlorothienopyrimidine intermediate 108, which enables nucleophilic aromatic substitution with a protected amine fragment to give compound 109. Subsequent amine displacement with methylamine installs the desired amino substituent, affording intermediate 110. Final acidic deprotection then delivers intermediate 111.

**Scheme 17:**
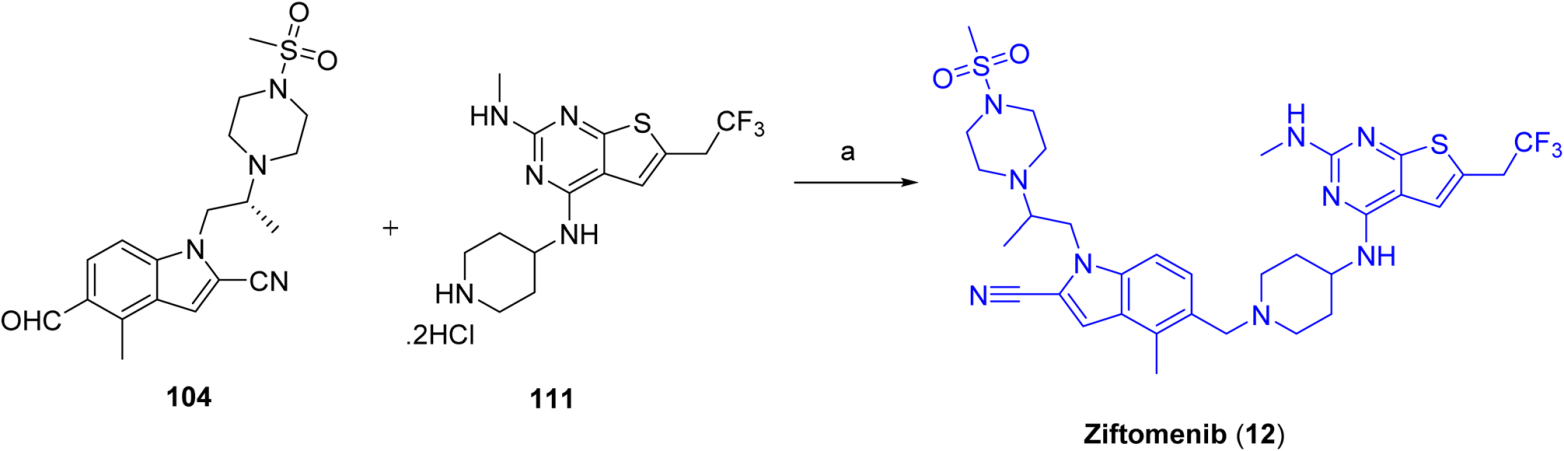
Synthesis of Ziftomenib (8). ^*a*^Reaction conditions: (a) isobutyric acid, NaBH_4_, DCM, TEA, 3 h.

Final assembly of ziftomenib (12) is shown in Scheme 17. A reductive amination strategy was used, in which an activated hydride system generated *in situ* from sodium borohydride and isobutyric acid mediated the reduction of a preassembled mixture of intermediates 104 and 111 in the presence of base, affording the final compound, ziftomenib (12).

### 2.13 Nuzolvence™ (zoliflodacin)

Nuzolvence™ (zoliflodacin, 13) is a first-in-class, orally bioavailable antibacterial agent with a spiropyrimidinetrione scaffold and a fluorinated benzisoxazole core, developed by Innoviva, Inc., for the treatment of uncomplicated urogenital *Neisseria gonorrhoeae* infections, including those caused by multidrug-resistant (MDR) strains. The drug received approval from the U.S. Food and Drug Administration on December 12, 2024, and *N. gonorrhoeae* is recognized as a priority pathogen by the Centers for Disease Control and Prevention and the World Health Organization. Zoliflodacin (13) exhibits bactericidal activity by selectively inhibiting bacterial type II topoisomerases, DNA gyrase, and topoisomerase IV, at a binding site distinct from that targeted by fluoroquinolones, thereby disrupting DNA replication and transcription. Zoliflodacin (13) was specifically designed to overcome key limitations of fluoroquinolones, including genotoxicity and bone marrow suppression (*e.g.*, anemia, leukopenia, and thrombocytopenia), while maintaining favorable pharmacokinetic and physicochemical properties.^52^*In vitro*, zoliflodacin (13) demonstrates potent activity against *N. gonorrhoeae*, with minimum inhibitory concentrations (MICs) ranging from ≤0.002 to 0.25 µg mL^−1^, and exhibits a broad antibacterial spectrum against other MDR Gram-positive, Gram-negative, and atypical pathogens. A limitation of zoliflodacin (13) is that its approved clinical use is currently restricted to uncomplicated gonococcal infections, and long-term postmarketing resistance and safety data remain limited. Its development represents a second-generation approach to DNA topoisomerase inhibition, offering a lower propensity for resistance development and an improved safety profile compared with existing fluoroquinolones therapies.^53^

**Scheme 18:**
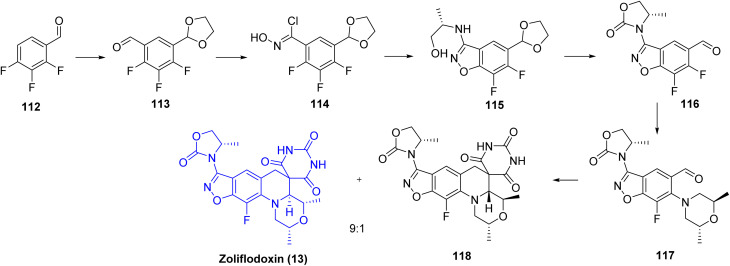
Synthesis of Zoliflodacin (13). ^*a*^Reaction conditions: (a) ethylene glycol, *p*-TsOH, refluxing toluene, 78% yield; (b) *n*-BuLi, −70 °C, THF, DMF quench, 93% yield; (c) NH_2_OH, EtOH, rt, 24 h, 80% yield; (d) NCS, DMF, rt, 4 h, 76% yield; (e) excess amine, DMF, rt, 1–3 h; (f) NaO*t*-Bu or Cs_2_CO_3_, rt, 2 steps, 59–93%; (g) CDI, DIEA, DMF, 70 °C, 2–3 h; (h) HCl, dioxane or THF, water, 70 °C, 2 steps: 75–99%; (i) (2R,6R)-2,6-dimethylmorpholine, K_2_CO_3_ or DIEA, CH_3_CN, water, 80 °C, 4–5 h, 73–98%; (j) pyrimidine-2,4,6(1H,3H,5H)-trione, AcOH, water 120 °C, 1h, 26–81%.

The synthesis of zoliflodacin (13) proceeds through the preparation of the key intermediate 116, obtained in four steps. The sequence begins with protection of aldehyde 112 to enable fluorine-directed *ortho*-lithiation,^54,55^ followed by quenching with DMF to furnish aldehyde 113. Conversion of 113 to the corresponding oximoyl chloride 114 is followed by chloride displacement with a chiral amino alcohol and subsequent base-promoted cyclization to afford the benzisoxazole intermediate 115. Formation of the oxazolidinone ring was achieved using CDI, and acidic hydrolysis of the acetal group provided aldehyde 116. Nucleophilic aromatic substitution of the fluoride adjacent to the aldehyde with dimethylmorpholine yielded intermediate 117, which was subjected to a Knoevenagel condensation followed by a T-reaction with barbituric acid, affording a 9 : 1 diastereomeric mixture dominated by the desired product zoliflodacin (13) (90%), with the remaining 10% corresponding to the minor diastereomer (118), Scheme 18.

### 2.14 Nereus™ (tradipitant)

Nereus™ (tradipitant, 14), is a selective antagonist of the human substance P/neurokinin-1 (NK-1) receptor. NK-1 receptors are widely expressed in the central nervous system, particularly within brainstem nuclei such as the nucleus tractus solitarius (NTS) and the area postrema, which integrate emetogenic inputs arising from vestibular and visceral stimuli, including motion-induced dizziness and vertigo. Peripheral expression of NK-1 receptors in the gastrointestinal tract further contributes to their role in emesis regulation. On December 30, 2025, tradipitant (14) received U.S. Food and Drug Administration approval for the prevention of motion-induced vomiting. This approval was supported by two randomized, double-blind, placebo-controlled Phase 3 clinical trials, Motion SYROS (NCT04327661) and Motion SERIFOS (NCT05903924). Beyond its antiemetic indication, tradipitant (14) has also been evaluated clinically for gastroparesis, COVID-19-associated pneumonia, and atopic dermatitis. Tradipitant (14) may impair mental and/or physical abilities needed for driving or operating machinery. Use with CNS depressants or strong CYP3A4 inhibitors may enhance this effect; if unavoidable, advise patients to avoid activities requiring full alertness.^56,57^

**Scheme 19:**
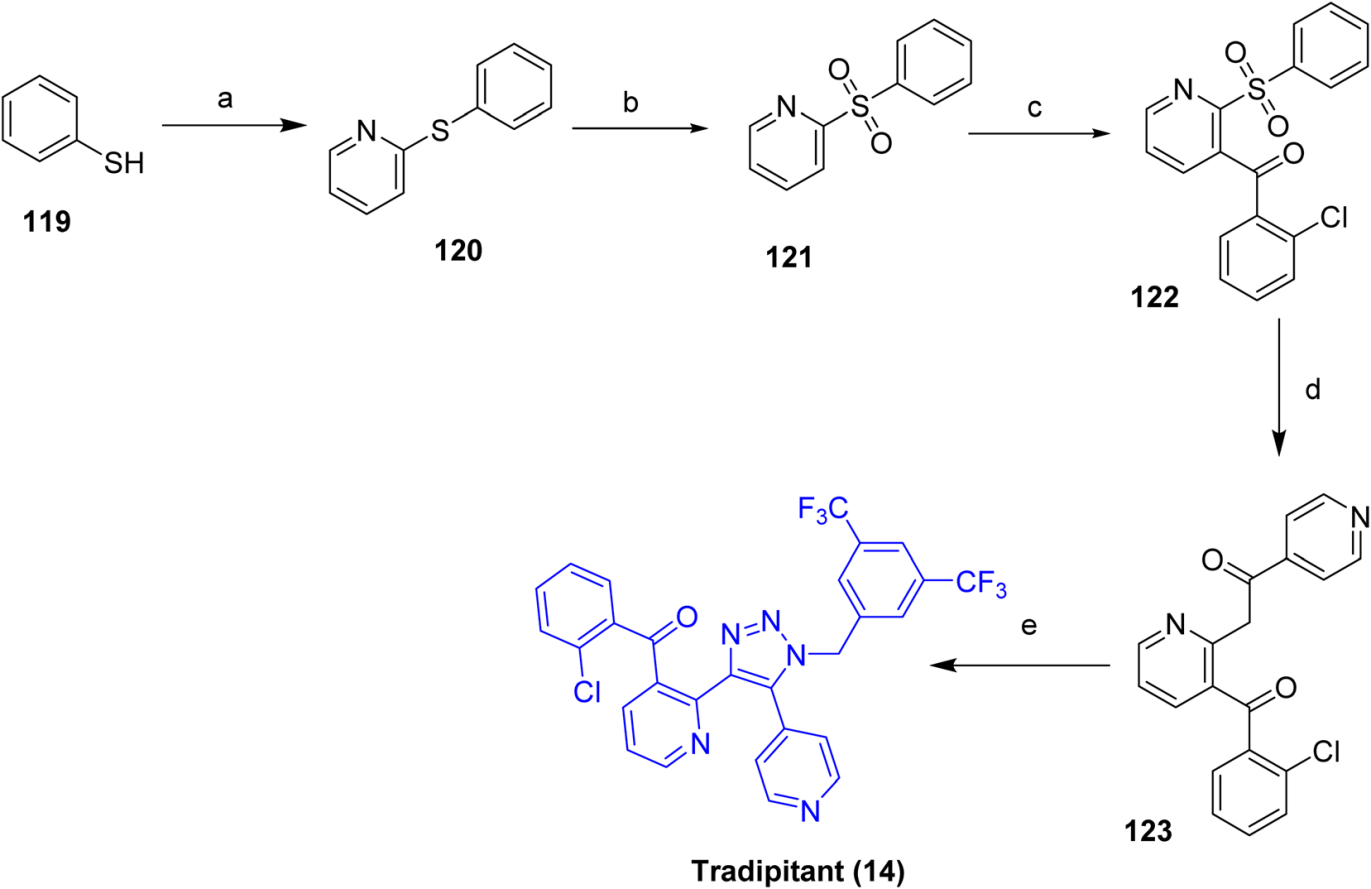
Synthesis of Tradipitant (14). ^*a*^Reaction conditions: (a) K_2_CO_3_, DMF at 110 °C; (b) acetic acid, sodium hypochlorite, DMF, 45 °C, 45 min; (c) *n-*BuLi, *i*-Pr_2_NH, 2-chlorobenzaldehyde, NaOCl, TEMPO; (d) 4-acetylpyridine, *t*-BuOK, DMSO, LiOH, PhCOOH, *i*PrOAc; (e) 3,5-bis(trifluoromethyl)benzyl azide, K_2_CO_3_, *t*-BuOH, 1 h at rt, then reflux for 18 h.

The synthesis of tradipitant (14) is outlined in Scheme 19.^58^ The sequence begins with nucleophilic substitution of 2-chloropyridine by thiophenol (119) under basic conditions to furnish the corresponding pyridyl sulfide (120), which is then oxidized to the sulfone intermediate 2-(benzenesulfonyl)pyridine (121). Directed lithiation of sulfone 121 with *n*-BuLi in the presence of diisopropylamine, followed by reaction with 2-chlorobenzaldehyde, affords the ketone intermediate 122. This ketone is then coupled with the enolate derived from 4-acetylpyridine, generated by treatment with *t*-BuOK in DMSO. Base-mediated cyclization in the presence of LiOH and benzoic acid yields the pyridine benzoate intermediate 123. In the final step, nucleophilic substitution with 3,5-bis(trifluoromethyl)benzyl azide provides the target compound, Tradipitant (14).

## 3 Summary of the drugs

Table 1 summarizes the key features of drugs approved by the U.S. Food and Drug Administration (FDA) in 2025, including the active pharmaceutical ingredients, therapeutic indications, recommended dosages, and approval dates.^12^

## 4 Conclusion

The 2025 evaluation of FDA-approved fluorinated drugs further underscores fluorine's pivotal role in contemporary medicinal chemistry and drug design. Of the 29 small-molecule drugs approved by the U.S. FDA in 2025, 14 contain fluorine, underscoring the continued use of fluorination strategies to meet demanding pharmacological and physicochemical requirements. The approved agents span a broad chemical space, with fluorine incorporated into diverse heterocyclic and aromatic frameworks, reflecting its versatility as a design element for optimizing potency, selectivity, metabolic stability, and bioavailability.

A dominant feature of the 2025 approvals is the extensive use of aromatic and heteroaromatic fluorination, particularly in kinase inhibitors and signal-transduction modulators such as Avmapki Fakzynja Co-Pack (avutometinib, 3a and defactinib, 3b), taletrectinib adipate (4), sunvozertinib (5), remibrutinib (10), and ziftomenib (12). In these molecules, fluorine substitution provides fine control over electronic properties, kinase selectivity, and resistance profiles, which are critical for mutation-driven and pathway-selective therapies. Trifluoromethyl and difluoromethyl motifs, as observed in agents such as imlunestrant (8), elinzanetant (11), and zoliflodacin (13), further highlight the strategic use of fluorine to modulate lipophilicity, binding affinity, and metabolic robustness.

Beyond oncology, fluorinated drugs approved in 2025 demonstrate significant therapeutic breadth, extending to pain management (suzetrigine, 1), immunology and inflammation (rilzabrutinib, 7), endocrinology and metabolic disorders (paltusotine, 9), infectious diseases (zoliflodacin, 13), and CNS-related indications (tradipitant, 14). Several of these compounds exhibit increased stereochemical complexity, consistent with evolving regulatory expectations for enantioselectivity, safety, and precise target engagement. This trend is particularly evident in fluorinated kinase inhibitors, BTK inhibitors, and CNS-active agents, where subtle stereochemical and electronic effects strongly influence efficacy and off-target liability.

Overall, the 2025 FDA approvals underscore the continued importance of fluorine chemistry in medicinal chemistry, demonstrating that fluorine incorporation remains a practical and effective strategy for optimizing molecular properties. These observations indicate that fluorine-containing motifs are likely to remain integral to future drug discovery efforts, particularly in the development of small molecules that require balanced potency, selectivity, and pharmacokinetic performance.

## Conflicts of interest

There are no conflicts of interest to declare.

## Data Availability

No primary research results, software, or code have been included, and no new data were generated or analyzed as part of this review.
